# Demystifying the *Capitella capitata* complex (Annelida, Capitellidae) diversity by morphological and molecular data along the Brazilian coast

**DOI:** 10.1371/journal.pone.0177760

**Published:** 2017-05-31

**Authors:** Camila F. Silva, Victor C. Seixas, Rômulo Barroso, Maikon Di Domenico, Antonia C. Z. Amaral, Paulo C. Paiva

**Affiliations:** 1 Departamento de Biologia Animal, Universidade Estadual de Campinas (UNICAMP), Campinas, SP, Brazil; 2 Departamento de Zoologia, Universidade Federal do Rio de Janeiro (UFRJ), Rio de Janeiro, RJ, Brazil; 3 Departamento de Biologia, Pontifícia Universidade Católica do Rio de Janeiro (PUC-RJ), Rio de Janeiro, RJ, Brazil; 4 Laboratório de Modelagem Ecológica, Centro de Estudos do Mar, Universidade Federal do Paraná, PR, Brazil; Laboratoire de Biologie du Développement de Villefranche-sur-Mer, FRANCE

## Abstract

The sibling species of *Capitella capitata* are globally known for their tolerance to disturbed habitats and the *C*. *capitata* complex is often used as an ecological indicator. A recent re-description proposed that *C*. *capitata*, originally described in Greenland is restricted to the Artic and Subarctic regions. Given their ecological relevance, we conducted a morphological and molecular analyses based on mtDNA sequences to investigate the diversity and distribution of the *C*. *capitata* complex along the Brazilian coast. Our morphological and molecular data were congruent and revealed the existence of four new species distinct from *C*. *capitata*, collected from the type locality. This study is the first characterization of the biodiversity and distribution of *Capitella* species made along the Brazilian coast and yielded a set of morphological characters corroborated by the mtDNA sequences for species identification. Our results increase the biodiversity of the genus along the Brazilian coast by describing four new species (*Capitella aracaensis* sp. n., *Capitella biota* sp. n., *Capitella neoaciculata* sp. n. and *Capitella nonatoi* sp. n.). One species was collected from only one sampling site, while the others are distributed along the coast.

## Introduction

The genus *Capitella* Blainville 1828 [1] (Capitellidae, Annelida) comprises polychaetes characterized by nine thoracic chaetigers with a species variation associated with the number of chaetigers bearing capillary chaetae and hooded hooks, genital spines present in chaetigers 8–9 of males and hermaphrodites, and the pygidium lacking appendages. There are 19 nominal species of *Capitella* described in a variety of environments (*e*.*g*., intertidal zone to abyssal depths) and habitats (*e*.*g*., soft-bottom, egg capsules, whale-bones) worldwide [2–5].

General attributes traditionally used to characterize capitellid species include overall size, shape and relative size of the prostomium and peristomium, formation of the peristomium as a complete or an incomplete ring, the number and distribution of capillary chaetae and hooded hooks along the thorax, the morphology of the genital spines, the number, size and structure of the hooded hooks and the shape of the pygidium [2–6]. However, distinguishing *Capitella* species is difficult and includes multiple complexes of cryptic species, which morphology alone has failed to define since the morphological features and life history traits can overlap.

The genus *Capitella* was the first marine invertebrate genus identified as a complex of cryptic species through molecular markers (allozyme) in the pioneer work of Grassle & Grassle [7], in which six different species, previously considered as *Capitella capitata*, were discovered along a short stretch of the USA Atlantic coast. Subsequent studies also identified cryptic species of *Capitella* through differences in their reproductive modes [8–9], gamete and larvae ultrastructure [10–11], developmental rates, dispersal patterns [12–14], adult body sizes [15] and physiological characteristics [16–17]. These criteria were used to define at least twelve cryptic species that were previously identified in laboratory cultures [3].

*Capitella capitata* [18] was originally described in Greenland, but the reports of its occurrence were later expanded to all oceans [3, 19–21]. The poor taxonomic understanding of the complex of cryptic species has led to broad distributions of these taxa, also known as ‘the cosmopolitan syndrome’ [22–23]. However, careful morphological revision of specimens and the advent of molecular techniques have increased the local diversity of several polychaete species and diminished the geographical distribution of species previously considered to be cosmopolitan [24–29].

After his re-description of *C*. *capitata* from the type locality, Blake [3] proposed this species is restricted to the Artic and Subarctic regions. Specimens of *C*. *capitata* that were recorded along the Brazilian coast [30–31] were therefor apparently misidentified. Moreover, the records of *C*. *capitata* from diverse benthic habitats and depths throughout the world indicate that a number of sibling and undescribed species are being overlooked taxonomically and ecologically.

*Capitella capitata* is referred to as an important ecological indicator, due to its high densities in polluted ecosystems [32–33] and as a model organism in many ecotoxicological studies [17, 34–35]. However, ecological studies are likely referring to an unknown number of species with different local and regional distributional patterns, toxicity tolerance [17], and reproductive strategies [34]. Given the ecological importance of this group the correct delimitation of species is essential. The aims of the present study were to investigate the diversity and distribution of the Brazilian populations of the *C*. *capitata* complex and characterize them morphologically and molecularly. Morphological characters and mtDNA sequences (COI and 16S) of specimens from 13 sites along the Brazilian coast were analyzed. Sequences from the type locality and public datasets were also included. The results will be useful in order to correctly identify the species in further distinct studies.

## Material and methods

### Study areas and sampling

**Fig 1:**
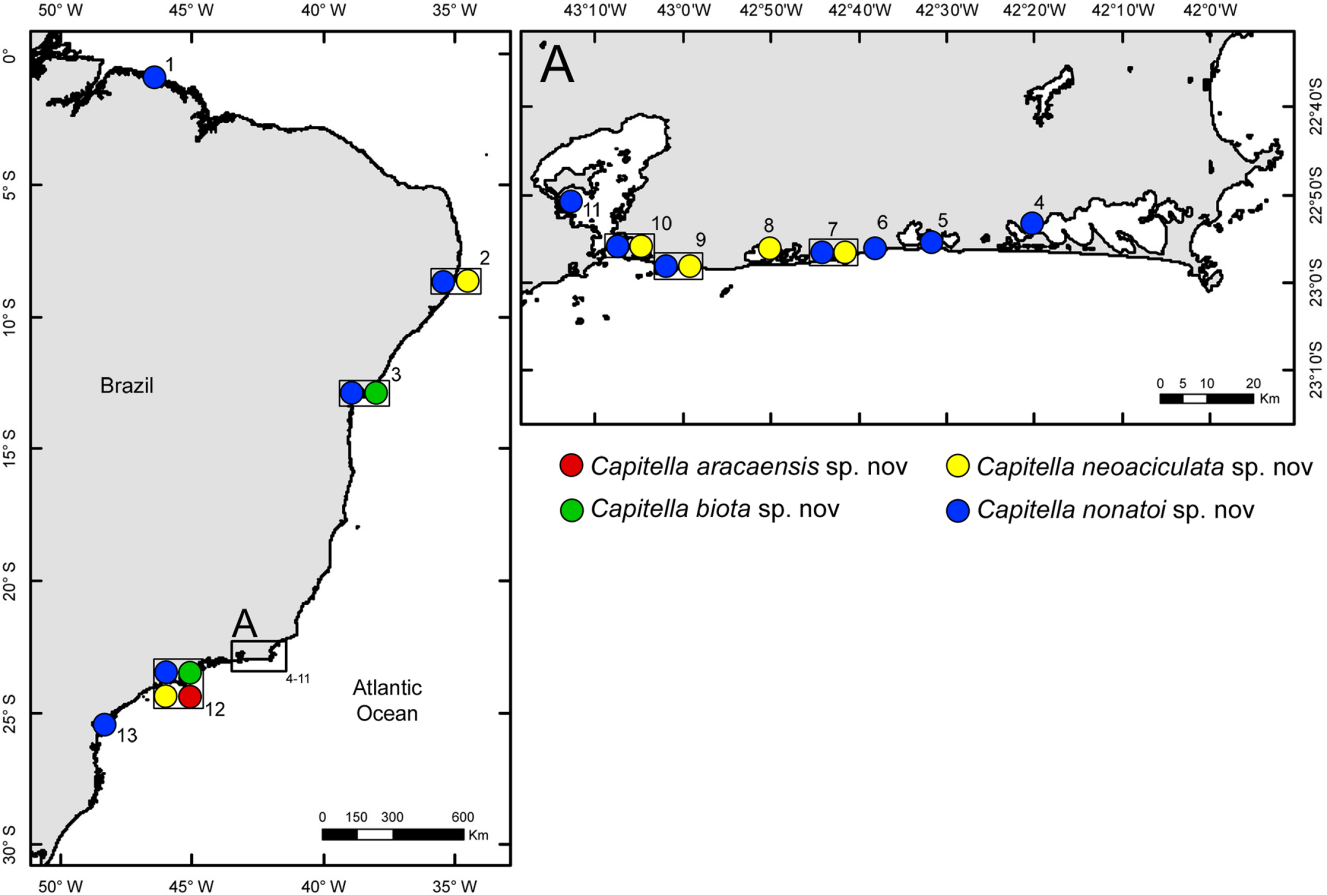
Distribution of *Capitella* species along the Brazilian coast. Circles within the same square indicate that more than one species was collected. Localities are numbered as follows: 1. Caetê Bay, Pará (0.49°S 46.36°W); 2. Maracaípe Mangrove, Pernambuco (8.31°S 35°W); 3. Todos os Santos Bay, Bahia (12.47°S 38.37°S); 4. Araruama Lagoon, Rio de Janeiro (22.53°S 42.23°W); 5. Saquarema Lagoon, Rio de Janeiro (22.55°S 42.33°W); 6. Jaconé Lagoon, Rio de Janeiro (22.56°S 42.39°W); 7. Guarapiná Lagoon, Rio de Janeiro (22.57°S 42.42°W); 8. Maricá Lagoon, Rio de Janeiro (22.55°S 42.49°W); 9. Itaipú Lagoon, Rio de Janeiro (22.57°S 43.2°W); 10. Piratininga Lagoon, Rio de Janeiro (22.57°S 43.5°W); 11. Guanabara Bay, Rio de Janeiro (22.5°S 43.13°W); 12. Araçá Bay, São Paulo (23.48°S 45.24°W); 13. Paranaguá Bay, Paraná (25.30°S 48.29°W).

Samples were collected from the intertidal zone and shallow waters (up to 0.5 m deep) at 13 sites along the Brazilian coast in six different states (Fig 1). Samples were also collected at one site in Greenland (02-Aug-2013; 69.25°S 54.10°W). Collected sediments were washed in the field on a 500 μm and 300 μm mesh sieve and any *Capitella* worms retained in the residue were fixed and preserved in 92% ethanol.

### Morphological analysis

Specimens were examined using optical microscopy (Zeiss Axio Imager M2), stereomicroscopy (Zeiss Axio Zoom v16) and, in some cases, with a Scanning Electron Microscope (SEM). Line drawings were made with a camera lucida attached to a compound microscope and afterwards hand drawn with Indian ink. Measurements were taken with an ocular micrometer that was calibrated with a stage micrometer. The body length was measured from the anterior margin of the prostomium to the tip of the pygidium, while the width was measured at the widest segment, excluding the chaetae. For SEM images, specimens were dehydrated in a series of ethanol solutions with progressively increasing concentrations (75–100%), critical-point-dried with a Balzers CPD 30 (temperature 37°C and pressure 70 kg/cm^2^), mounted on stubs, covered with a layer of 10–20 nm of gold, and observed under the SEM at the Laboratório de Microscopia Eletrônica, Instituto de Biologia, Universidade Estadual de Campinas (UNICAMP) using the JEOL JSM-5800 LV Scanning Electron Miscroscope.

The nomenclature used for chaetal morphology follows that usually used for Capitellidae species and some suggested by Green [2]. The chaetal characters used here are:

- main fang: format and angle with the hook shaft;
- teeth: number of teeth and rows that are arranged above the main fang;
- shoulder: development of the curvature of the hook;
- hood: size, format, size of the opening and texture;
- anterior shaft: length between shoulder and node;
- node: presence or absence and shape;
- posterior shaft: length between node and the end of the hook.

The material was deposited at the Museu de Zoologia, Universidade Estadual de Campinas (ZUEC), São Paulo, Brazil and the Museu Nacional do Rio de Janeiro (MNRJ), Rio de Janeiro, Brazil.

### Nomenclatural acts

The electronic edition of this article conforms to the requirements of the amended International Code of Zoological Nomenclature, and hence the new names contained herein are available under that Code from the electronic edition of this article. This published work and the nomenclatural acts it contains have been registered in ZooBank, the online registration system for the ICZN. The ZooBank LSIDs (Life Science Identifiers) can be resolved and the associated information viewed through any standard web browser by appending the LSID to the prefix “http://zoobank.org/”. The LSID for this publication is: urn:lsid:zoobank.org:pub: 7C72C6E6-F616-4A60-87D7-C2239F03AB14. The electronic edition of this work was published in a journal with an ISSN, and has been archived and is available from the following digital repositories: PubMed Central, LOCKSS.

### Molecular analysis

#### DNA extraction, amplification and sequencing

Genomic DNA was extracted with a DNeasy Blood & Tissue Kit (QIAGEN) and in some cases according to the protocol in Floyd et al. [36]. Fragments of the mitochondrial gene, cytochrome oxidase subunit 1 (COI), were amplified using universal primers LCO1490 and HCO2198 [37] or degenerated primers degLCO1490 and degHCO2198 [38]. For the 16S ribosomal DNA (16S) the universal primers 16Sar-L and 16Sbr-H [39] were used. PCR reactions for both loci consisted of PuReTaq Ready-To-Go™ PCR Beads (GE Healthcare), 1.5 μL of each primer (0.6 mM), 2 μL of DNA and 20 μL of water. The thermal cycling conditions for COI were one cycle of 94°C for 3 min, 5 cycles of 94°C for 40 s, 47°C for 40 s and 72°C for 1 min, 32 cycles of 94°C for 40 s, 52°C for 40 s and 72°C for 1 min, followed by a final extension step of 72°C for 5 min. For the 16S amplification, the reaction consisted of one cycle of 94°C for 3 min, 5 cycles of 94°C for 30 s, 42°C for 40 s and 72°C for 90 s, 32 cycles of 94°C for 30 s, 46°C for 40 s and 72°C for 90 s, followed by a final extension step of 72°C for 7 min. The resulting PCR products were purified and sequenced by Macrogen, Inc.

#### Data analysis

Electropherograms were edited with Sequencher 4.1 (Gene Codes Corporation), and sequences were aligned with MAFFT 7.0 [40] using the G-INS-I strategy for COI and Q-INS-I for 16S. COI sequences were checked for translation using the invertebrate mitochondrial genetic code. As saturation was not detected in the saturation test [41], all codon positions were used, as implemented in DAMBE5 [42]. The phylogenetic analysis was performed using the maximum likelihood (ML) algorithm and Bayesian inference (BI). Three datasets were considered, COI, 16S and concatenated (COI+16S). The ML trees were estimated in RAxML 8.2 [43] using the substitution models GTR+G for all datasets. The best ML tree was obtained from 20 initial independent trees, and the statistical support was obtained with a rapid bootstrap function (-f a) using 1000 replicates. The BI was conducted in MrBayes 3.2 [44] under the HKY+I+G model for COI and 16S, and HKY+G for the concatenated dataset, as selected in the jModelTest 2 [45] using Bayesian information criterion (BIC). Tree parameters were sampled every 1000 generations, for a total of 10^7^ samples. Two independent runs and 4 chains were implemented. The final results were checked according to the standard deviations of split frequencies (<0.01). The effective sampling size (ESS>200) was assessed via Tracer 1.5 [46]. The ML and BI analyses were conducted in CIPRES Science Gateway [47]. Species from two other Capitellidae genera, *Notomastus* (16S and COI) and *Heteromastus* (COI), were considered as outgroups and were used to root the trees. The concatenated dataset used only *Notomastus* as an outgroup as no 16S sequences are available for *Heteromastus*. Intra- and interspecific pair-wise genetic distances were estimated under the *p*-distance and Kimura 2 parameters models with MEGA 6.1 [48]. GenBank sequences of *C*. *capitata* from other localities were taken to compare with our sequences. Data collection of specimens, museum codes and GenBank (www.ncbi.nlm.nih.gov/genbank) accession numbers are detailed in Table 1.

**Table 1:** Collection information for specimens used in morphological and molecular analyses and GenBank accession numbers.

		Morphology		Molecular			
Species	Sample site	SPEC. #		COI	GenBank ID COI	16S	GenBank ID 16S
***Capitella neoaciculata* sp. n.**	Maracaípe Mangrove, PE (8.31°S 35°W)		.	3	KX121855-KX121857	.	.
	Guarapiná Lagoon, RJ (22.57°S 42.42°W)		.	3	KX121828-KX121830	3	KX121957-KX121959
	Maricá Lagoon, RJ (22.55°S 42.49°W)		.	5	KX121831-KX121835	5	KX121960-KX121964
	Itaipú Lagoon, RJ (22.57°S 43.2°W)	45	ZUEC POL: 17648	5	KX121836-KX121840	5	KX121965-KX121969
	Piratininga Lagoon, RJ (22.57°S 43.5°W)	34	ZUEC POL: 17647	5	KX121841-KX121845	5	KX121970-KX121974
	Araça Bay, SP (23.48°S 45.24°W)	655	ZUEC POL: 16786–16825 / 17373–17436. MNRJP: 1429	9	KX121846-KX121854	14	KX121975-KX121988
***Capitella aracaensis* sp. n.**	Araça Bay, SP (23.48°S 45.24°W)	33	ZUEC POL: 16779 / 17437–17458. MNRJP: 994	3	KX121876-KX121878	4	KX122007-KX122010
***Capitella biota* sp. n.**	Todos os Santos Bay, BA (12.47°S 38.37°S)		.	10	KX121866-KX121875	9	KX121998-KX122006
	Araça Bay, SP (23.48°S 45.24°W)	152	ZUEC POL: 16728–16729 / 16731–16778. MNRJP: 997	8	KX121858-KX121865	9	KX121989-KX121997
***Capitella nonatoi* sp. n.**	Caetê Bay, PA (0.49°S 46.36°W)	11	ZUEC POL: 17652	.	.	3	KX122011-KX122013
	Maracaípe Mangrove, PE (8.31°S 35°W)		.	1	KX121827	1	KX121956
	Todos os Santos Bay, BA (12.47°S 38.37°S)		.	9	KX121818-KX121826	11	KX121945-KX121955
	Araruama Lagoon, RJ (22.53°S 42.23°W)		.	5	KX121773-KX121777	5	KX121880-KX121884
	Saquarema Lagoon, RJ (22.55°S 42.33°W)		.	5	KX121778-KX121782	5	KX121885-KX121889
	Jaconé Lagoon, RJ (22.56°S 42.39°W)		.	5	KX121783-KX121787	5	KX121890-KX121894
	Guarapiná Lagoonn, RJ (22.57°S 42.42°W)		.	5	KX121802-KX121806	5	KX121895-KX121899
	Itaipú Lagoon, RJ (22.57°S 43.2°W)	15	ZUEC POL: 17650	5	KX121788-KX121792	5	KX121900-KX121904
	Piratininga Lagoon, RJ (22.57°S 43.5°W)		.	4	KX121793-KX121796	5	KX121905-KX121909
	Guanabara Bay, RJ (22.5°S 43.13°W)		.	5	KX121797-KX121801	5	KX121910-KX121914
	Araçá Bay, SP (23.48°S 45.24°W)	3479	ZUEC POL: 17459–17473 / 17581–17649. MNRJP: 995–996	7	KX121811-KX121817	21	KX121915-KX121935
	Paranaguá Bay, PR (25.30°S 48.29°W)	2	ZUEC POL: 17651	4	KX121807-KX121810	9	KX121936-KX121944
*Capitella capitata*	Greenland (69.25°N 54.10°W)		.	1	KX121879	1	KX122014
	Indo-Pacific			4	JX676137, JX676150, JX676171, JX676179	.	.
	Hudson Bay, Canada			6	HQ023469-HQ023473, GU672407	.	.
*Capitella* cf. *Capitata*	Gulf of Mexico, Galveston, USA			2	KX961404, KX961414	.	.
	Gulf of Mexico, Florida, USA			2	KX961432, KX961427	.	.
*Capitella* cf. *Aciculata*	Gulf of Mexico, Galveston, USA			2	KX961408. KX961411	.	.
	Gulf of Mexico, Florida, USA			2	KX961424, KX961433	.	.
*Capitella teleta*	Miyagi, Japan			3	LC120627, LC120631, LC120638	1	JF509722
*Capitella* aff. *teleta*	Miyagi, Japan			3	LC120644, LC120646, LC120650	.	.
*Capitella* sp.	Hokkaido, Japan			1	LC120652	.	.
*Notomastus profundus*				1	KR916897	.	.
*Notomastus* sp.				.	.	1	KF511858
*Notomastus hemipodus*				.	.	1	HM746714
*Heteromastus filiformis*		* *	* *	2	KR916852-KR916853		

^a^ PE, Pernambuco

^b^ RJ, Rio de Janeiro

^c^ SP, São Paulo

^d^ BA, Bahia

^e^ PA, Pará

^f^ PR, Paraná.

^g^ SPEC.

^h^ COI, cytochrome oxidase subunit 1

^i^ 16S, ribosomal DNA

^j^ ZUEC POL, Museu de Zoologia da Universidade Estadual de Campinas

^k^ MNRJ, Museu Nacional do Rio de Janeiro.

# specimen number

## Results

### Morphological analysis

Examination of 4,423 specimens from different localities along the Brazilian coast allowed us to classify them into four species within *Capitella*: 33 *C*. *aracaensis* sp. n., 149 *C*. *biota* sp. n., 734 *C*. *neoaciculata* sp. n., and 3,507 *C*. *nonatoi* sp. n. These species were identified mainly by the overall size, shape and size of the prostomium and peristomium, formation of the peristomium as a complete or an incomplete ring, the number and distribution of capillary chaetae and hooded hooks along the thoracic chaetigers, the details of the genital spines, the number, size and structure of the hooded hooks and the shape of the pygidium. The differences of these characteristics among the *Capitella* species were already summarized in a table by Silva et al. [5]. Here, we provide the description of the species and a key to the new species among all valid *Capitella* species.

### Taxonomic account

Family Capitellidae Grube, 1862 [49]

Genus *Capitella* Blainville, 1828 [1]

Type species. *Capitella capitata* (Fabricius, 1780) as *Lumbricus capitatus*. [18] Redescribed by Blake, 2009 [3].

Type locality. West Greenland.

#### Diagnosis (emended after Magalhães & Bailey-Brock, 2012 [4])

Prostomium conical to bluntly rounded, sometimes dorsoventrally flattened, with a dorsal groove present or absent, with nuchal organs as paired slits at border between prostomium and peristomium; eyespots present or absent. Peristomium forming a complete or an incomplete achaetous ring. Thorax with 10 segments including an achaetous peristomium and nine chaetigers. Capillary chaetae in both rami of chaetigers 1–3, 1–4, 1–6, or 1–7 or capillaries and hooks in various combinations in both rami, and chaetigers 8–9 with hooded hooks, mixed capillaries and hooks, or all capillaries; arrangements sometimes size dependent. Genital hooks present in chaetigers 8–9 of males and hermaphrodites; females usually with enlarged lateral genital pores between chaetigers 7–8 or 8–9. Capillaries unilimbate, hooded hooks with multiple rows of denticles above the main fang. Abdominal segments with hooded hooks in both rami, without capillaries. Branchiae present or absent, and pygidium without appendages.

### Key to all valid species of *Capitella*

**1a.** Capillary chaetae on chaetigers 1 – 3: **2**

**1b.** Capillary chaetae on chaetigers 1 – 4: **3**

**1c.** Capillary chaetae on chaetigers 1 – 5: **4**

**1d.** Capillary chaetae on chaetigers 1 – 6: **5**

**1e.** Capillary chaetae on chaetigers 1 – 7: **6**

**1f.** Capillary chaetae on chaetigers 1 – 8: **7**

**2a.** Mixed chaetae and hooded hooks in noto- and neuropodia of chaetigers 4 – 7; hooded hooks on chaetigers 8 and 9; eyespots present: *C*. *capitata tripartita* Hartman 1961 [50]

**2b.** Hooded hooks on chaetigers 4 – 9; prostomium equitriangular; peristomium forming an incomplete achaetous ring; eyespots present; genital spines present; hooded hooks with three teeth above main fang in a single row: *C*. *jonesi* (Hartman 1959) [51]

**2c.** Hooded hooks on chaetigers 4 – 9; prostomium conical; peristomium forming a complete achaetous ring; eyespots absent; genital spines present; hooded hooks with 7 − 9 teeth above main fang arranged in three rows: *C*. *minima tulearensis* (Thomasin 1970) [52]

**2d.** Hooded hooks on chaetigers 4 – 9; prostomium conical; peristomium forming a complete achaetous ring; eyespots absent; genital spines present; thoracic hooded hooks with one apical tooth and abdominal hooks with two teeth above main fang, one above the other: ***C*. *biota* sp. n.**

**3a.** Mixed chaetae and hooded hooks in noto- and neuropodia of chaetigers 5 – 7; hooded hooks on chaetigers 8 and 9; prostomium conical; peristomium forming a complete achaetous ring; eyespots absent: *C*. *ovincula* Hartman 1947 [20]

**3b.** Hooded hooks on chaetigers 5 – 9; prostomium equitriangular; peristomium forming an incomplete achaetous ring; eyespots absent; hooded hooks with four teeth above main fang in a single row: *C*. *capitata floridana* Hartman 1959 [51]

**3c.** Hooded hooks on chaetigers 5 – 9; prostomium triangular; peristomium forming a complete achaetous ring; eyespots absent; hooded hooks with 5 − 6 teeth above main fang arranged in two rows: *C*. *minima* Langerhans 1881 [53]

**3d.** Hooded hooks on chaetigers 5 – 9; prostomium conical; peristomium forming a complete achaetous ring; eyespots present; hooded hooks with 10 teeth above main fang arranged in two rows: *C*. *hermaphrodita* Boletzky & Dohle 1967 [54]

**4a.** Modified chaetae in noto-and neuropodia of chaetigers 6 – 9; prostomium conical; peristomium forming a complete achaetous ring; eyespots absent: *C*. *aberranta* Hartman & Fauchald 1971 [55]

**5a.** Hooded hooks on chaetigers 7 – 9; prostomium conical: *C*. *gracilis* (Verrill 1880) [56]

**5b.** Hooded hooks on chaetigers 7 – 9; prostomium conical; peristomium forming an incomplete achaetous ring; eyespots absent; hooded hooks with 10 − 12 teeth above main fang arranged in four rows: *C*. *giardi* (Mesnil 1897) [57]

**6a.** Hooded hooks in notopodia and mixed chaetae and hooded hooks in neuropodia of chaetigers 8 and 9; prostomium conical; peristomium forming an incomplete achaetous ring; eyespots absent; hooded hooks with one small tooth above main fang: *C*. *perarmata* (Gravier 1911) [58]

**6b.** Hooded hooks on chaetigers 8 and 9; prostomium short and conical, flattened dorsoventrally; peristomium forming a complete achaetous ring; eyespots absent; thoracic chaetigers rugose; abdominal chaetigers with dark brown pigmented dorsum; hooded hooks with two rows of teeth above main fang: *C*. *amboensis* Pamunkgas 2017 [59]

**6c.** Hooded hooks on chaetigers 8 and 9; prostomium conical; peristomium forming a complete achaetous ring; eyespots absent; thoracic and abdominal hooded hooks with two teeth above main fang, one above the other: ***C*. *aracaensis* sp. n.**

**6d.** Hooded hooks on chaetigers 8 and 9; prostomium short and rounded with a mid-ventral depression; peristomium forming an incomplete achaetous ring; eyespots absent; hooded hooks with numerous teeth above main fang arranged in five rows: *C*. *capitata* (Fabricius 1780) [18]

**6e.** Hooded hooks on chaetigers 8 and 9; prostomium conical with a dorsal depression and a ventral groove; peristomium forming a complete achaetous ring; eyespots present in juveniles; hooded hooks with numerous teeth above main fang arranged in four rows: *C*. *caribaeorum* Warren & George 1986 [60]

**6f.** Hooded hooks on chaetigers 8 and 9; prostomium conical; peristomium forming an incomplete achaetous ring; eyespots absent; hooded hooks with four teeth above main fang in a single row: *C*. *dizonata* Johnson 1901 [61]

**6g.** Hooded hooks on chaetigers 8 and 9; prostomium rounded; peristomium forming a complete achaetous ring; eyespots absent; thoracic hooded hooks with 5 − 6 teeth above main fang arranged in two rows; abdominal hooded hooks with 8 − 9 teeth above main fang arranged in three rows: *C*. *iatapiuna* Silva et al. 2016 [5]

**6h.** Hooded hooks on chaetigers 8 and 9; prostomium rounded with a dorsal smooth depression and ventral groove; peristomium forming an incomplete achaetous ring; eyespots present; thoracic hooded hooks with six teeth above main fang arranged in two rows; abdominal hooded hooks with three teeth above main fang arranged in two rows: ***C*. *nonatoi* sp. n.**

**6i.** Hooded hooks on chaetigers 8 and 9; prostomium conical; peristomium forming a complete achaetous ring; eyespots absent; hooded hooks with 11 − 14 teeth above main fang arranged in two rows; branchiae present: *C*. *singularis* (Fauvel 1932) [62]

**6j.** Hooded hooks on chaetigers 8 and 9; prostomium triangular; peristomium forming an incomplete achaetous ring; eyespots present; hooded hooks with six teeth above main fang arranged in two rows: *C*. *teleta* Blake et al. 2009 [6]

**7a.** Hooded hooks on chaetiger 9: *C*. *teres* (Treadwell 1939) [63]

**7b.** Acicular spines in noto- and neuropodia of chaetigers 1 and 2 of males and generally in notopodia of chaetiger 1 of females; prostomium triangular with smooth dorsal depression and ventral groove; peristomium forming an incomplete achaetous ring; eyespots absent; thoracic and abdominal hooded hooks with 5 teeth above main fang arranged in two rows: *C*. *aciculata* (Hartman 1959) [51]

**7c.** Acicular spines in noto- and neuropodia of chaetigers 1 and 2 of males and capillary chaetae on females; prostomium triangular with smooth dorsal depression and ventral groove; peristomium forming an incomplete achaetous ring; eyespots absent; thoracic and abdominal hooded hooks with 6 teeth above main fang arranged in two rows, pygidium large, heart-shaped: ***C*. *neoaciculata* sp. n.**

#### *Capitella aracaensis* sp. n. Silva & Amaral Figs 2–4

urn:lsid:zoobank.org:act:1B37F3BF-EB6C-4E57-8C9F-677B8AA92F43.

**Fig 2:**
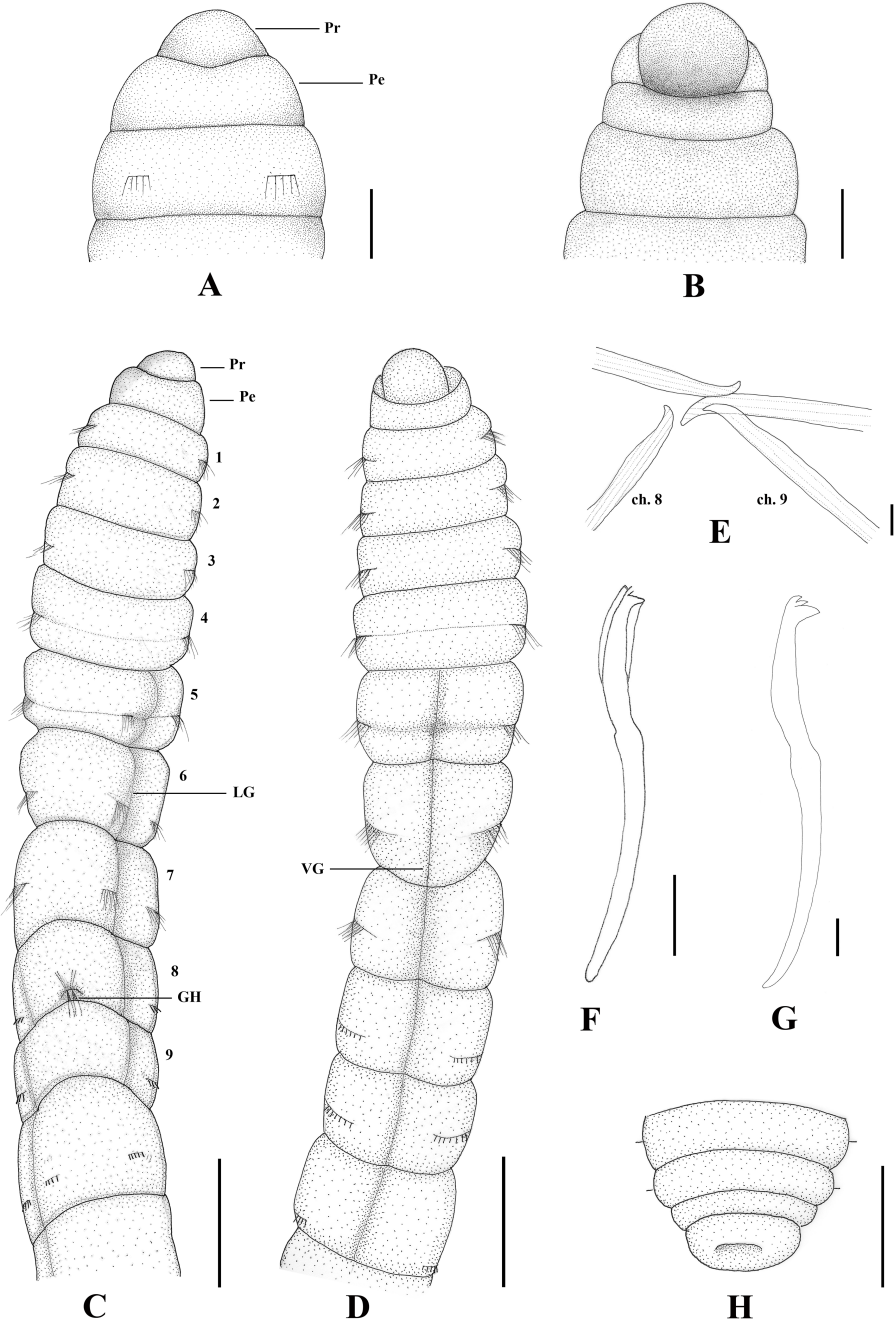
*Capitella aracaensis* sp. n. (A) Anterior end, dorsal view; (B) Anterior end, ventral view; (C) Thoracic region, dorsal view; (D) Thoracic region, ventral view; (E) Genital spines; (F) Thoracic hooded hook, lateral view; (G) Abdominal hooded hook, lateral view; (H) Pygidium, dorsal view. Ch: chaetiger. GH: genital hook. LG: lateral groove. Pe: peristomium. Pr: prostomium. VG: ventral groove. Scale bars: A, B, 0.1 mm; C, D, 0.3 mm; E, F, 20 μm; G, 10 μm; H, 0.125 mm.

**Fig 3:**
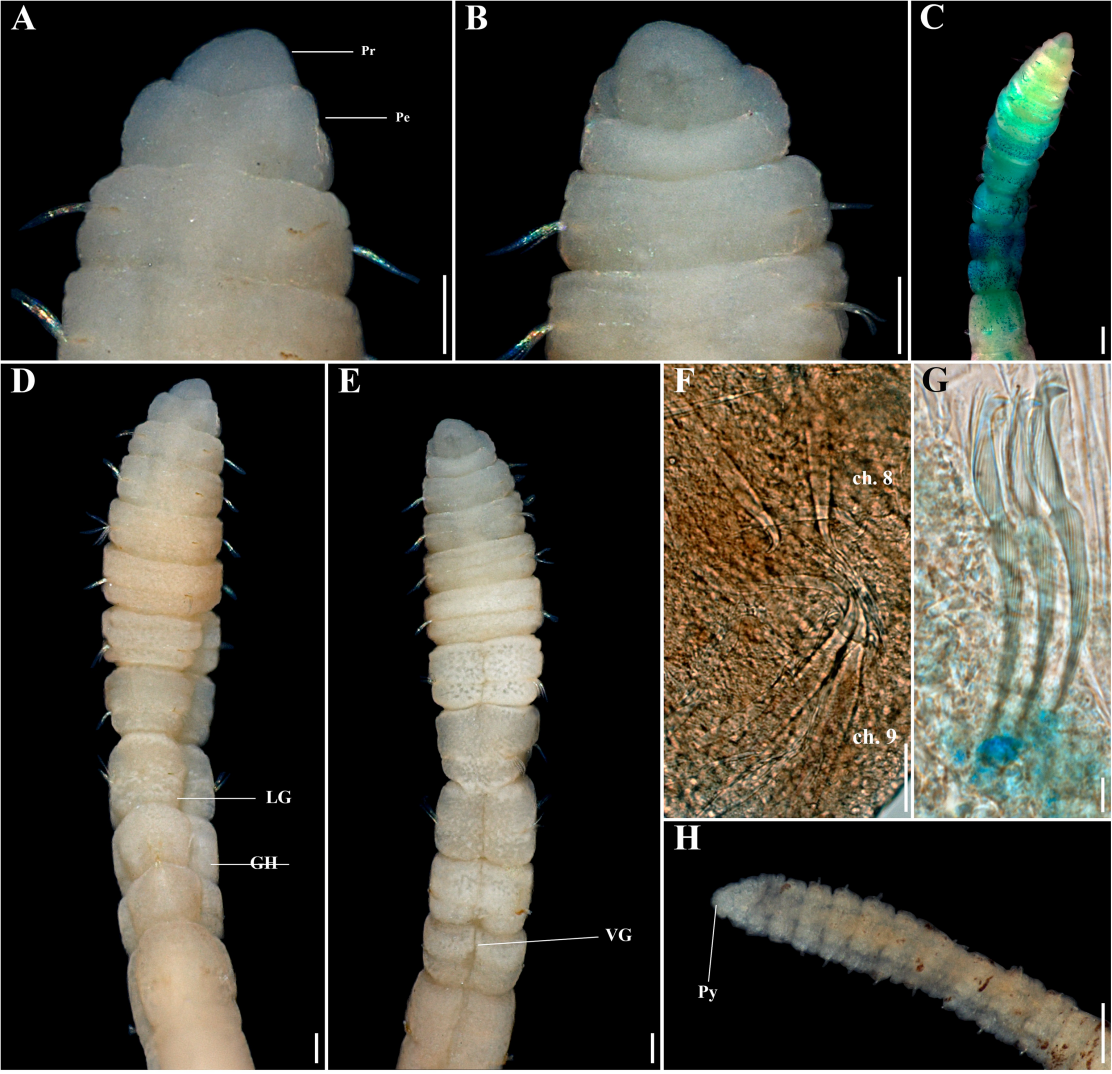
*Capitella aracaensis* sp. n. (A) Anterior end, dorsal view; (B) Anterior end, ventral view; (C) Methyl green staining pattern; (D) Thoracic region, dorsal view; (E) Thoracic region, ventral view; (F) Genital spines; (G) Abdominal hooded hook, lateral view; (H) Posterior end and pygidium. Ch: chaetiger. GH: genital hook. LG: lateral groove. Pe: peristomium. Pr: prostomium. Py: pygidium. VG: ventral groove. Scale bars: A, B, D, E, F, H, 0.1 mm; C, 0.2 mm; G, 10 μm.

**Fig 4:**
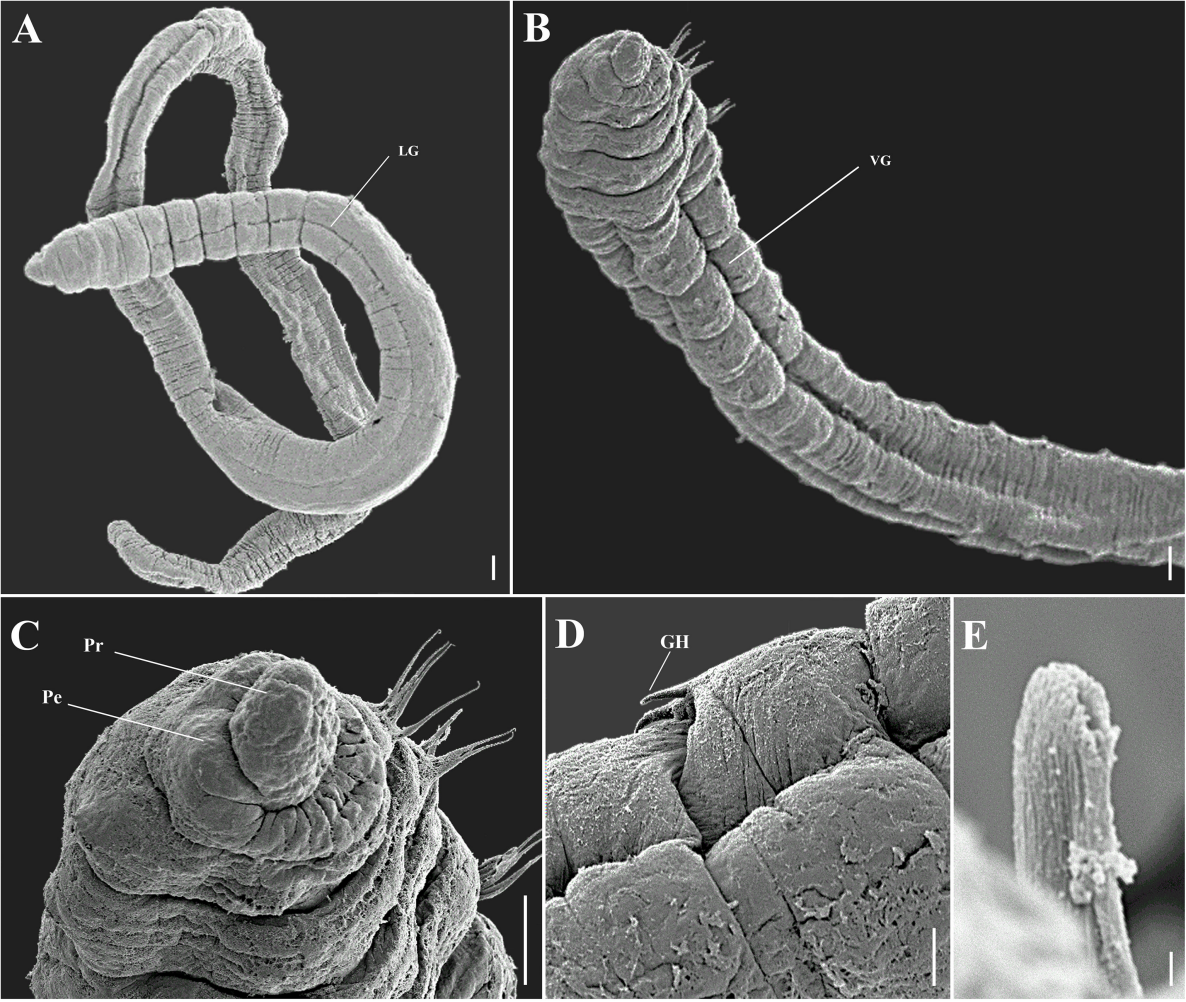
*Capitella aracaensis* sp. n., SEM. (A) Complete specimen; (B) Thoracic region, ventral view; (C) Anterior end, latero-ventral view; (D) Genital spines; (E) Abdominal hooded hook, frontal view. GH: genital hook. LG: lateral groove. Pe: peristomium. Pr: prostomium. VG: ventral groove. Scale bars: A, B, C, 0.1 mm; D, 0.5 mm; E, 1 μm.

**Holotype:** São Paulo, Araçá Bay: ZUEC POL 16779: 23°48'55,1"S − 45°24'25,9"W; tidal flat; station 79(2)A; 07 May 2012; 1 spec.

**Paratypes:** São Paulo, Araçá Bay: ZUEC POL 17451 –paratype 1: 23°48'50,7"S − 45°24'28,0"W; tidal flat; station 51(3)A; 24 Feb 2012; 1 spec. ZUEC POL 17457 –paratypes 2 − 6: 23°48'36,1"S − 45°24'19,5"W; tidal flat; station 34(3)A; 29 Sep 2011; 5 specs. MNRJP 994 –paratype 7: 23°48'55,1"S − 45°24'25,9"W; tidal flat; station 79(2)A; 07 May 2012; 1 spec.

**Additional material examined (S1 Appendix):** São Paulo, Araçá Bay (25 specs.).

**Description.** Based on type material, additional material and specimens examined by SEM. Size range of material examined (complete individuals) 11.37–15.44 mm long, 0.45–0.6 mm wide and 50–62 chaetigers. Specimens slightly widest anteriorly, gradual narrowing posteriorly. Color in alcohol brownish. Prostomium conical, wider than longer (Figs 2A and 2C; 3A and 3D). Peristomium large, forming a complete achaetous ring, conspicuous dorsal and ventrally, wider than peristomium (Figs 2A and 2B; 3A and 3B); eyespots absent. Nuchal organs not visible using light microscopy or SEM. Chaetigers 1–4 similar, rectangular, weakly biannulate; chaetigers 5–9 similar, square, with mid-ventral and lateral groove (Figs 2C and 2D; 3D and 3E; 4A and 4B). Male and female adult specimens with unilimbate capillaries in notopodia and neuropodia of chaetigers 1–7, hooded hooks in neuropodia of chaetigers 8–9 and genital spines in notopodia of chaetigers 8–9. Notosetae arranged in a single row of 3–7 capillaries and 5–6 hooded hooks; neurosetae arranged in a single row of 3–8 capillaries and 4–6 hooded hooks. Thoracic hooded hooks with pointed, straight and short main fang, right angle with the shaft, surmounted by 2 apical teeth, one above the other; long curved shoulder; anterior shaft absent; developed node; long and slightly curved posterior shaft; short and smooth hood (Fig 2F). Chaetigers 8 and 9 with two straight genital spines with tips sharply curved and thin vertical grooves; spines of chaetiger 8 embedded and shorter than those of chaetiger 9; spines of chaetiger 9 external and larger than those of chaetiger 8 (Figs 2E; 3F and 4D). Division between thorax and abdomen not prominent (Figs 2C and 2D; 3D and 3E; 3A and 3B). Abdominal chaetigers as long as wide (Figs 2C and 3D); chaetigers with 8–9 hooded hooks in notopodia and 9–11 in neuropodia, reduced to one hook in far posterior; hooks slightly smaller than the thoracics with pointed, straight and short main fang, right angle with the shaft, protruding just slightly through frontal opening, surmounted by 2 teeth, one above the other; long and curved shoulder; anterior shaft absent; well-developed node; long and curved posterior shaft; long and smooth hood (Figs 2G; 3G and 4E). Branchiae absent. Pygidium a quite small simple lobe without anal cirri (Figs 2H and 3H).

**Methyl green staining pattern.** Chaetigers 5–7 with a strip of small spots in the middle of the segment, chaetigers 8 and 9 darkly stained and abdominal segments staining uniformly (Fig 3C).

**Biology.** All specimens with genital spines. However, a few specimens presented oocytes inside the abdominal region, confirming they are female specimens.

**Remarks.** *Capitella aracaensis* sp. n., belongs to a group of species of *Capitella* with capillary chaetae on chaetigers 1–7 and hooded hooks on chaetigers 8 and 9. This group includes *C*. *amboensis*, *C*. *capitata*, *C*. *caribaeorum*, *C*. *dizonata*, *C*. *iatapiuna*, *C*. *perarmata*, *C*. *singularis* and *C*. *teleta*. *Capitella aracaensis* sp. n. shares some features with *C*. *caribaeorum*, *C*. *iatapiuna* and *C*. *singularis*, such as the peristomium forming a complete ring and the absence of eyespots. These species differ, however, in the characteristics of their prostomium: in *C*. *caribaeorum* it is conical, with a dorsal depression and ventral groove; in *C*. *iatapiuna* it is quite rounded, as long as wide; and in *C*. *singularis* it is conical and smooth; while in *C*. *aracaensis* sp. n. it is rounded. The abdominal hooded hooks also differ in number and distribution of teeth above main fang: in *C*. *caribaeorum* there are several teeth arranged in four rows; in *C*. *iatapiuna* there are 5 − 6 teeth arranged in two rows on thoracic hooks and 8 − 9 teeth in three rows on abdominal hooks; and in *C*. *singularis* there are 11 − 14 teeth arranged in two rows; while in *C*. *aracaensis* sp. n. there are two teeth, one above the other, on both thoracic and abdominal hooded hooks. *Capitella capitata*, *C*. *dizonata*, *C*. *perarmata* and *C*. *teleta* differ from *C*. *aracaensis* sp. n. by having a peristomium forming an incomplete ring and in features of the hooded hooks. *Capitella aracaensis* sp. n. can be distinguished by its rounded prostomium, large complete peristomium and hooded hooks with two teeth above the main fang, one above the other.

**Etymology.** This species was named after the Araçá Bay (São Sebastião, state of São), which has a high biodiversity [64] and is one of the samplings areas of this study.

**Habitat.** Intertidal region, in fine sand and mangrove.

**Type locality.** Aracá Bay, São Sebastião, São Paulo, Brazil (South Atlantic Ocean).

**Distribution.** South Atlantic Ocean: Brazil (state of São Paulo).

#### *Capitella biota* sp. n. Silva & Amaral Figs 5 and 6

urn:lsid:zoobank.org:act:66EA9584-1C4C-4074-A735-624A73806F72.

**Fig 5:**
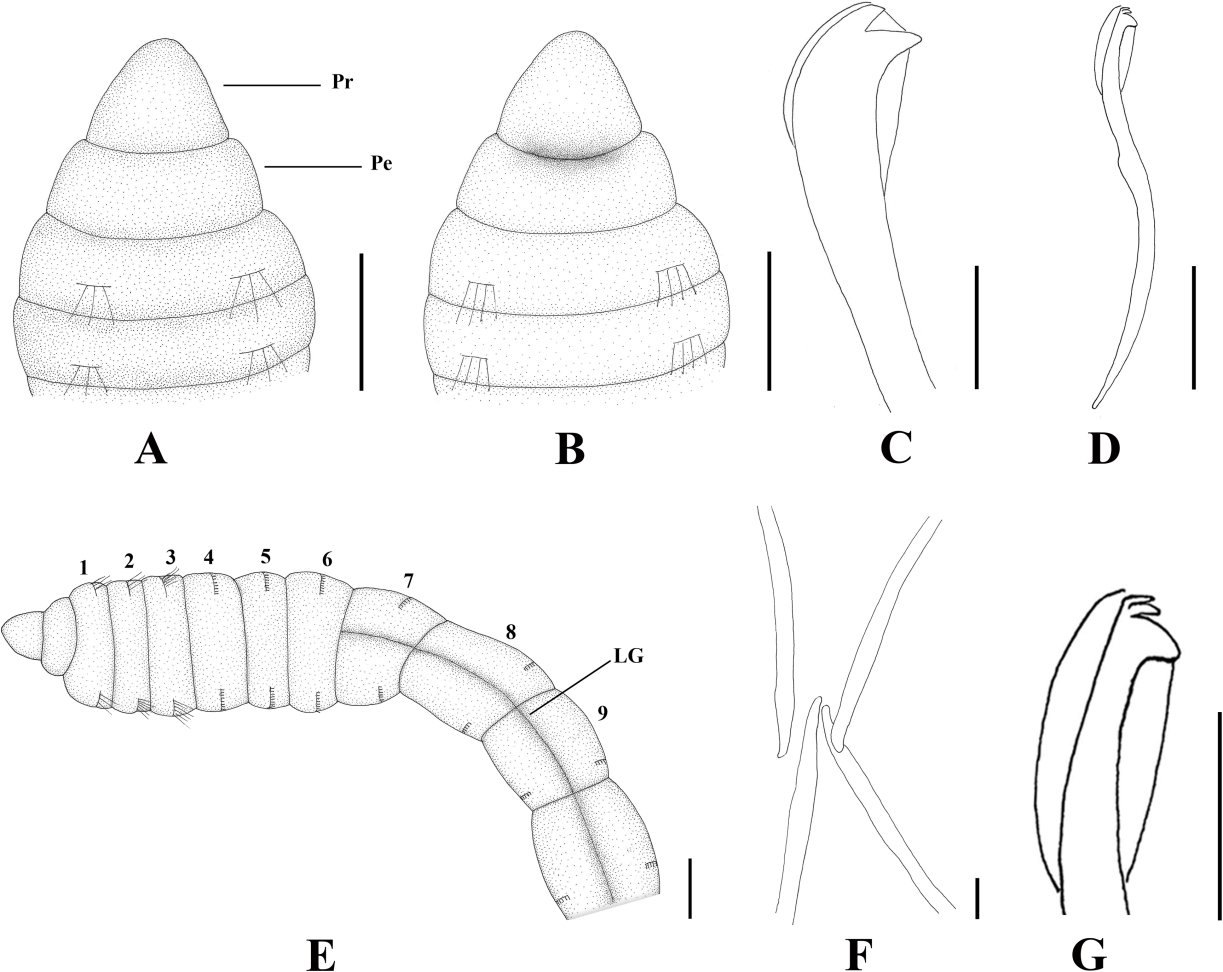
*Capitella biota* sp. n. (A) Anterior end, dorsal view; (B) Anterior end, ventral view; (C) Thoracic hooded hook, lateral view; (D) Abdominal hooded hook, lateral view; (E) Thoracic region, lateral view; (F) Genital spines; (G) Abdominal hooded hook, anterior end. Scale bars: A, B, E, 0.1 mm; C, 5 μm; D, F, 15.6 μm; G, 7.8 μm.

**Fig 6:**
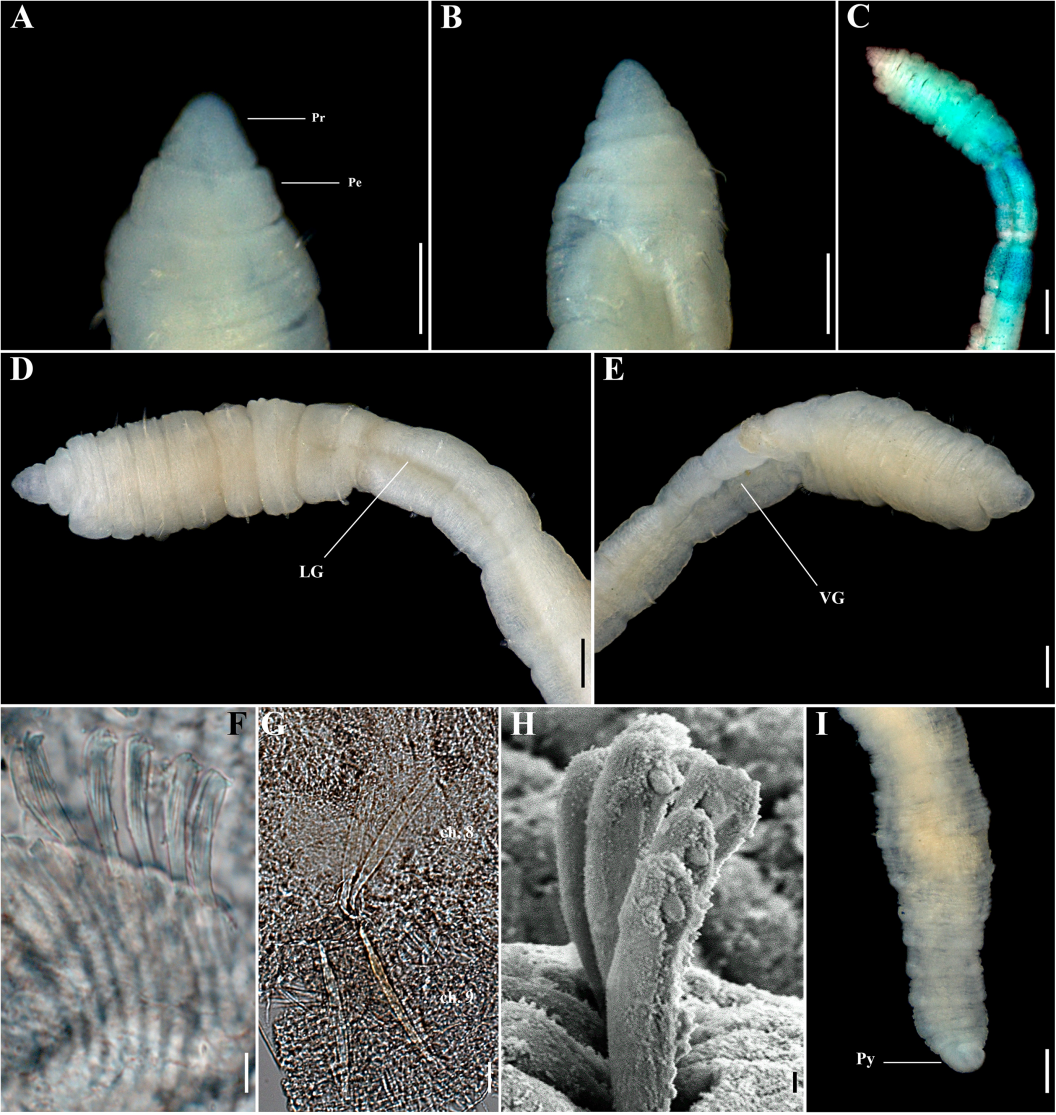
*Capitella biota* sp. n. (A) Anterior end, dorsal view; (B) Anterior end, ventral view; (C) Methyl green staining pattern; (D) Thoracic region, lateral view; (E) Thoracic region, ventral view; (F) Thoracic hooded hooks, lateral view; (G) Genital spines; (H) Abdominal hooded hooks, frontal view; (I) Posterior end and pygidium. Pe: peristomium. Pr: prostomium. Py: pygidium. Scale bars: A, B, D, E, I, 0.1 mm; C, 0.2 mm; F, 5 μm; G, 20 μm; H, 1 μm.

**Holotype:** São Paulo, Araçá Bay: ZUEC POL 16728: 23°48'51,4''S − 45°24'26,5''W; mangrove; station 62M; coll. 19 Mar 2014; 1 spec.

**Paratypes:** São Paulo, Araçá Bay: ZUEC POL 16729 –paratype 1: 23°48'51,4''S − 45°24'26,5''W; mangrove; station 62M; coll. 19 Mar 2014; 1 spec. MNRJP 997 –paratypes 2 − 3: 23°48'51,4''S − 45°24'26,5''W; mangrove; station 63M; coll. 19 Mar 2014; 2 specs. ZUEC POL 16731 –paratype 4: 23°48'51,4''S − 45°24'26,5''W; mangrove; station 144M; coll. 10 Jul 2014; 1 spec. ZUEC POL 16732 –paratypes 5 − 7: 23°48'37,4"S − 45°24'21,4"W; tidal flat; station 117(4); coll. 17 Sep 2013; 3 specs.

**Additional material examined (S1 Appendix):** São Paulo, Araçá Bay (141 specs.).

**Description.** Based on type material, additional material and specimens examined by SEM. Size range of material examined (complete individuals) 3.9–16.0 mm long (holotype 16.0 mm), 0.31–0.42 mm wide (holotype 0.4 mm) and 30–74 chaetigers (holotype 74 chaetigers). Body small, widest anteriorly, gradual narrowing posteriorly. Color in alcohol yellowish. Prostomium pointed, longer than wider (Figs 5A and 5B; 6A and 6B). Peristomium distinct, forming a complete achaetous ring, conspicuous dorsal- and ventrally, wider than prostomium (Figs 5A and 5B; 6A and 6B); eyespots absent. Chaetigers 1–7 similar; chaetigers 8 and 9 slightly more narrow; all chaetigers with shallow intersegmental furrows; chaetigers 7–9 with a deep mid-ventral groove (Figs 5D and 6E) and a smooth lateral groove (Fig 6D). Adult specimens with unilimbate capillaries in notopodia and neuropodia of chaetigers 1–3 and hooded hooks in notopodia and neuropodia of chaetigers 4–9. Notosetae arranged in a single row of 3–7 capillaries and 7–8 hooded hooks; neurosetae arranged in a single row of 3–8 capillaries and 5–9 hooded hooks; chaetae emerging from the middle of the chaetigers (Figs 5D and 6D). Thoracic hooded hooks with pointed and short main fang, upward curved, surmounted by one apical tooth; long and slightly curved shoulder; long and smooth hood (Fig 6F). Division between thorax and abdomen not prominent (Figs 5D; 6D and 6E). Abdominal chaetigers as long as wide, anterior chaetigers with 6–9 hooded hooks in notopodia and 6–10 in neuropodia, reducing to 2–3 hooks in posterior chaetigers, emerging from the last third of the chaetigers. Hooded hooks small with rounded and robust main fang, right angle with the shaft, protruding through the frontal opening, surmounted by two teeth, one above the other; long and slightly curved shoulder; anterior shaft absent; developed node; long and well curved posterior shaft; long and smooth hood (Figs 5F; 6H). Chaetigers 8 and 9 with 2 embedded genital spines. Spines of chaetiger 8 thin, straight and with slightly curved tips; spines of chaetiger 9 larger than those of chaetiger 8, straight and with slightly curved tips (Figs 5E; 6G). Branchiae absent. Pygidium small simple lobe without anal cirri (Fig 6I).

**Methyl green staining pattern.** Chaetigers 7 − 9 and the first abdominal chaetiger darkly stained (Fig 6C).

**Remarks.** *Capitella biota* sp. n., belongs to a group of species of *Capitella* with capillary chaetae on chaetigers 1–3. This group includes *C*. *capitata tripartita*, *C*. *jonesi* and *C*. *minima tulearensis*. Although *C*. *jonesi* and *C*. *minima tulearensis* also have hooded hooks in noto- and neuropodia of chaetigers 4 − 9, the former has a peristomium forming an incomplete ring and eyespots, while the latter has hooded hooks with 7 − 9 teeth above main fang, distributed in three rows, both differing from *C*. *biota* sp. n. *Capitella capitata tripartita* differs from *C*. *biota* sp. n. in having eyespots and mixed capillary chaetae and hooded hooks in noto- and neuropodia of chaetigers 4–9. *Capitella biota* sp. n. can be distinguished by having a peristomium forming a complete ring and thoracic hooded hooks with one apical tooth and abdominal hooks with two teeth above main fang, one above the other.

**Etymology.** This species was named after the “BIOTA–FAPESP Program”, which allowed the realization of the “BIOTA–Araçá Project”, responsible for funding the collection of most of the individuals reported in this paper.

**Habitat.** Intertidal region, in fine sand and mangrove sediments.

**Type locality.** Aracá Bay, São Sebastião, São Paulo, Brazil (South Atlantic Ocean).

**Distribution.** South Atlantic Ocean: Brazil (states of Bahia and São Paulo).

#### *Capitella neoaciculata* sp. n. Silva & Seixas Figs 7–9

urn:lsid:zoobank.org:act:138592D0-D7E3-4C5D-9DB4-6F2E009A73E9.

**Fig 7:**
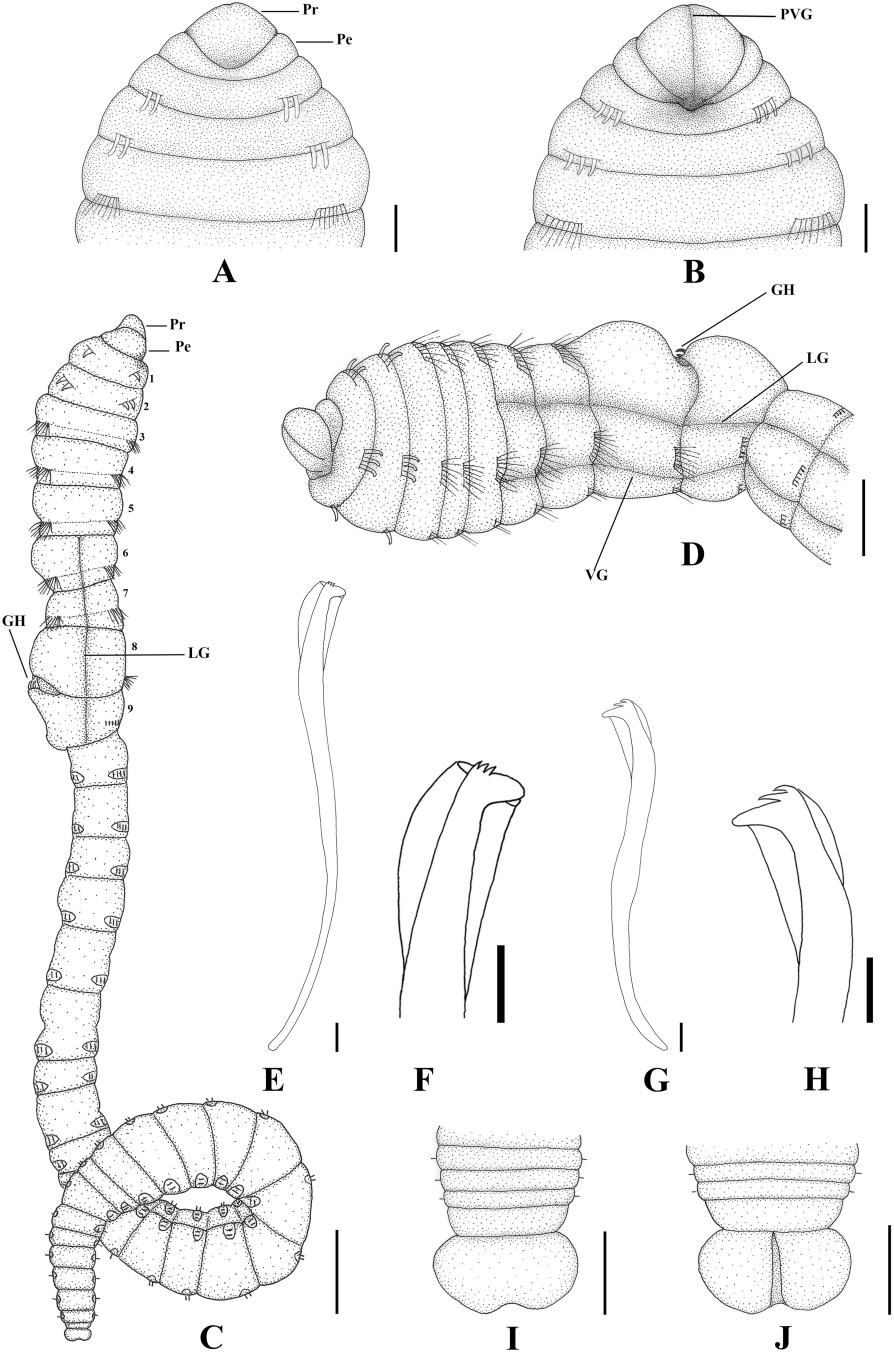
*Capitella neoaciculata* sp. n. (A) Anterior end, dorsal view; (B) Anterior end, ventral view; (C) Complete male specimen, lateral view; (D) Thoracic region of a male, lateral view; (E) Thoracic hooded hook, lateral view; (F) Thoracic hooded hook, anterior end; (G) Abdominal hooded hook, lateral view; (H) Abdominal hooded hook, anterior end; (I) Pygidium, dorsal view; (J) Pygidium, ventral view. GH: genital hook. LG: lateral groove. Pe: peristomium. Pr: prostomium. PVG: prostomial ventral groove. VG: ventral groove. Scale bars: A, B, D, 0.2 mm; C, 0.5 mm; E, F, G, H, 5 μm; I, J, 0.125 mm.

**Fig 8:**
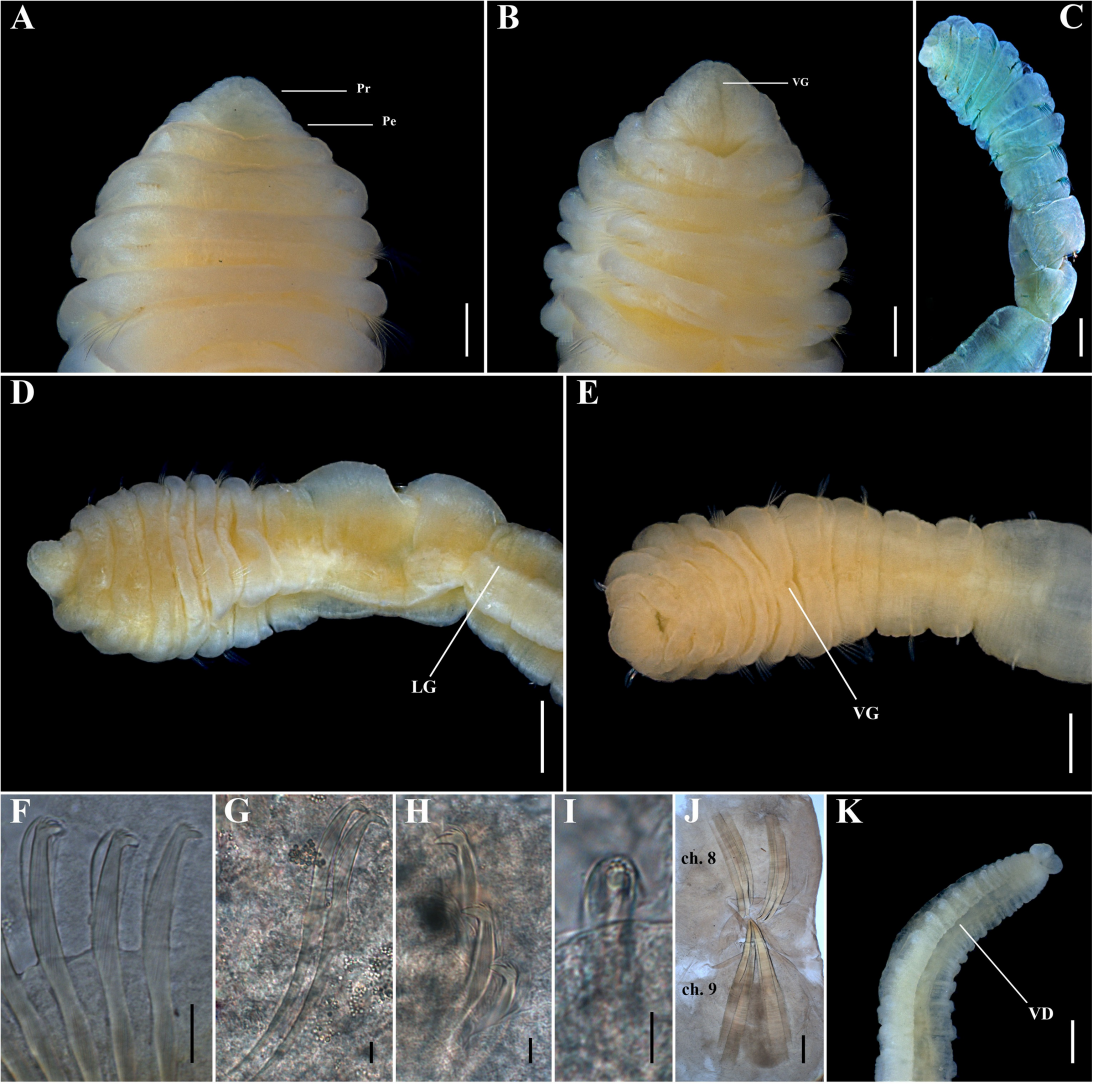
*Capitella neoaciculata* sp. n. (A) Anterior end, dorsal view; (B) Anterior end, ventral view; (C) Methyl green staining pattern; (D) Thoracic region of a male, lateral view; (E) Thoracic region of a male, ventral view; (F) Thoracic hooded hook, lateral view; (G, H) Abdominal hooded hook, lateral view; (I) Abdominal hooded hook, frontal view; (J) Genital spines; (K) Posterior end and pygidium. Ch: chaetiger. LG: lateral groove. Pe: peristomium. Pr: prostomium. VD: ventral depression. VG: ventral groove. Scale bars: A, B, D, E, K, 0.2 mm; C, 0.5 mm; F, G, H, I, 5 μm; J, 0.1 mm.

**Fig 9:**
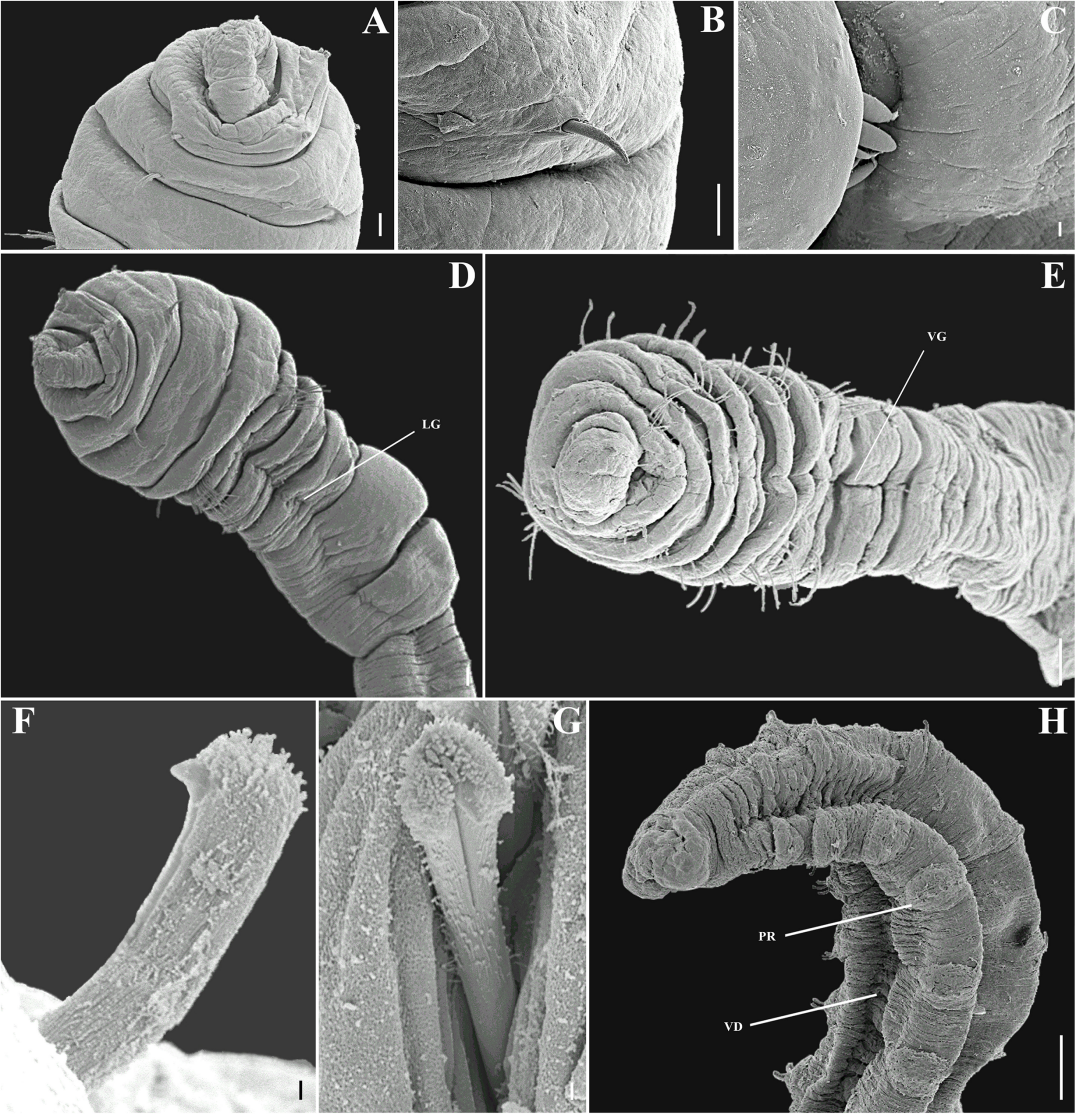
*Capitella neoaciculata* sp. n., SEM. (A) Anterior end, lateral view; (B) Acicular spine; (C) Genital spines; (D) Thoracic region of a male, lateral view; (E) Thoracic region of a male, ventral view; (F) Thoracic hooded hook, lateral view; (G) Abdominal hooded hook, frontal view; (H) Posterior end and pygidium. LG: lateral groove. PR: parapodial ridges. VD: ventral depression. VG: ventral groove. Scale bars: A, B, D, E, H, 0.1 mm; C, 10 μm; F, G, 1 μm.

**Holotype:** São Paulo, Araçá Bay: ZUEC POL 16816: 23°48'51,4"S − 45°24'26,5"W; mangrove; station 205M; coll. 17 Mar 2015; 1 spec.

**Paratypes:** São Paulo, Araçá Bay: ZUEC POL 17399: 23°48'46,6''S − 45°24'29,8''W; mangrove; station 100M; coll. 10 Jul 2014; 2 specs. ZUEC POL 17384: 23°48'51,4''S − 45°24'26,5''W; mangrove; station 137M; coll. 10 Jul 2014; 1 spec. MNRJ 1429: 23°48'51,4"S − 45°24'26,5"W; mangrove; station 205M; coll. 17 Mar 2015; 6 specs.

**Additional material examined (S1 Appendix):** São Paulo, Araçá Bay (655 specs.); Rio de Janeiro, Piratininga Lagoon (34 specs.); Rio de Janeiro, Itaipu Lagoon (45 specs.).

**Description.** Based on type material, additional material and specimens examined by SEM. Size range of material examined (complete individuals) 5.0–22.0 mm long, 0.2–1.2 mm wide and 30–73 chaetigers. Specimens widest anteriorly, gradual narrowing posteriorly (Fig 7C). Color in alcohol yellowish. Prostomium triangular, wider than longer, with a deep dorsal depression (Figs 7A and 7C; 8A; 9A and 9D) and a slight ventral groove (Figs 7B; 8B and 9E). Peristomium forming an incomplete achaetous ring, slightly conspicuous dorsal and laterally (Figs 7A, 7C and 7D; 8B, 8D and 8E; 9A); similar in width to the first chaetiger; eyespots absent. Chaetiger 1 the smallest; chaetigers 2 − 4 and 7 similar in width and length; chaetigers 5 and 6 wider and longer; chaetigers 8 and 9 rectangular in female, rounded in male; all chaetigers with shallow intersegmental furrows; chaetigers 5 − 9 with mid-ventral and lateral groove (Figs 7D; 8D and 8E; 9D and 9E). Adult female specimens with capillaries in notopodia and neuropodia of chaetigers 1 − 8, hooded hooks in notopodia and neuropodia of chaetiger 9, and a pair of genital spines in neuropodia of chaetiger 9. Male adult specimens with acicular spines in notopodia and neuropodia of chaetigers 1 and 2 (Figs 7A–7D; 8C; 9A and 9D), capillaries in notopodia and neuropodia of chaetigers 3 − 7, capillaries in neuropodia of chaetiger 8, hooded hooks in neuropodia of chaetiger 9, and genital spines in notopodia of chaetigers 8 and 9. Noto- and neuroaciculae arranged in a single row of 2 or 3 acicular spines; noto- and neurosetae arranged in a single row of 5 − 13 unilimbate capillaries and 6 − 10 hooded hooks; chaetae emerging from the last third of the chaetiger. Thoracic hooded hooks with a rounded, thick and slightly curved main fang, surmounted by six apical teeth arranged in two rows (3 basally and 3 in superior row); long straight shoulder; short anterior shaft; inconspicuous node; long posterior shaft; long and smooth hood (Figs 7E and 7F; 8F and 9F). In females, chaetiger 9 with a pair of small, thin and slightly curved genital spines. In males, chaetiger 8 with 6 falcate external genital spines (2 fascicles) with tips sharply curved, narrower than those of chaetiger 9; chaetiger 9 with 6 straight embedded genital spines (2 fascicles), curved apically, with blunt tips, larger than those of chaetiger 8 (Figs 8J and 9C). Division between thorax and abdomen prominent. Abdominal chaetigers as long as wide; chaetigers with 3 − 10 hooded hooks in notopodia and 5 − 15 in neuropodia, reducing to 4 notopodial and 7 neuropodial hooks. Abdominal hooded hooks shorter than the thoracics with a pointed, thin and slightly curved main fang, surmounted by six teeth arranged in two rows (3 basally and 3 on superior row); long and slightly curved shoulder, short anterior shaft; developed node; long posterior shaft; short and smooth hood (Figs 7G and 7H; 8G, 8H and 8I and 9G). Ventral depression along the abdominal region (Figs 8K and 9H). Neuropodial hooded hooks emerging from long parapodial ridges in far posterior chaetigers (Figs 8K and 9H). Branchiae absent. Pygidium a large simple lobe, fused dorsally and slightly bilobate ventrally (heart-shaped), without anal cirri (Figs 7I and 7J; 8K and 9H).

**Methyl green staining pattern.** Specimens staining uniformly, except the two first chaetigers which are lightly speckled (Fig 8C).

**Variation.** The presence of the acicular spines varies according to the length and perhaps sexual development of the specimens. Large females (more than 0.8 mm wide) can have acicular spines in notopodia of chaetiger 1, however the aciculae are less developed than in males; and small males, with genital spines less developed, commonly lack acicular spines in neuropodia of chaetigers 1 and 2. Larger specimens can also have capillary chaetae on chaetiger 9.

**Biology.** Almost all specimens with genital spines; specimens with small and less developed genital spines only on chaetiger 9 presented oocytes inside the abdominal region, confirming they are female specimens. Males, with robust genital spines, did not present oocytes.

**Remarks.** *Capitella neoaciculata* sp. n., belongs to a group of species of *Capitella* with capillary chaetae on chaetigers 1–8 and hooded hooks on chaetiger 9. This group includes *C*. *aciculata* and *C*. *teres*. *Capitella neoaciculata* sp. n. differs from *C*. *teres* by having acicular spines in the first chaetigers. Besides the presence of acicular spines, *Capitella neoaciculata* sp. n. differs from *C*. *aciculata*, in that females lack acicular spines, in the notopodia of chaetigers 1 and 2, hooded hooks have six teeth arranged in two rows (3 basally and 3 on superior row), rather than five teeth (3 basally and 2 on superior row), there is a ventral depression along the abdominal region, and the pygidium is large and heart-shaped rather than inconspicuous and a simple ring as in *C*. *aciculata*.

**Etymology.** This species was named based on the presence of acicular spines as *Capitella aciculata*, however, with a new combination of characters.

**Habitat.** From intertidal region to shallow subtidal regions (up to 0.5 m), in fine sand.

**Type locality.** Aracá Bay, São Sebastião, São Paulo, Brazil (South Atlantic Ocean).

**Distribution.** South Atlantic Ocean: Brazil (states of São Paulo, Rio de Janeiro and Pernambuco).

#### *Capitella nonatoi* sp. n. Silva & Amaral Figs 10–12

urn:lsid:zoobank.org:act:6F87DE88-1079-41A9-8A1C-780006A46538.

**Fig 10:**
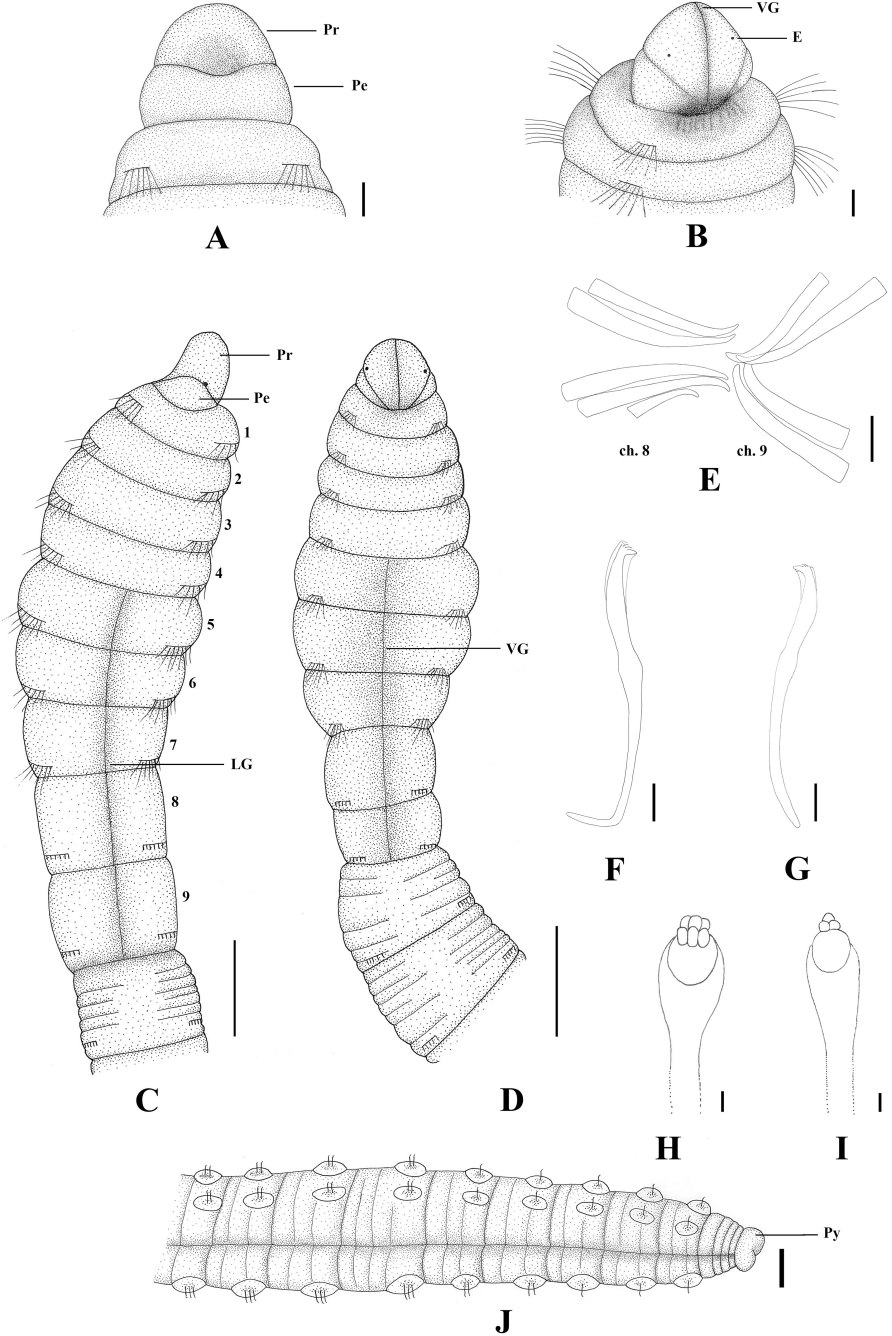
*Capitella nonatoi* sp. n. (A) Anterior end, dorsal view; (B) Anterior end, ventral view; (C) Thoracic region of a female specimen, lateral view; (D) Thoracic region of a female specimen, ventral view; (E) Genital hooks; (F) Thoracic hooded hook, lateral view; (G) Abdominal hooded hook, lateral view; (H) Thoracic hooded hook, frontal view; (I) Abdominal hooded hook, frontal view; (J) Posterior end with pygidium, dorso-lateral view. E: eyespot. LG: lateral groove. Pe: peristomium. Pr: prostomium. PVG: prostomial ventral groove. Py: pygidium. VG: ventral groove. Scale bars: A, B, 0.01 mm; C, D, 1 mm; E, 0.125 mm; F, G, 10 μm; H, I, 1 μm; J, 0.1 mm.

**Fig 11:**
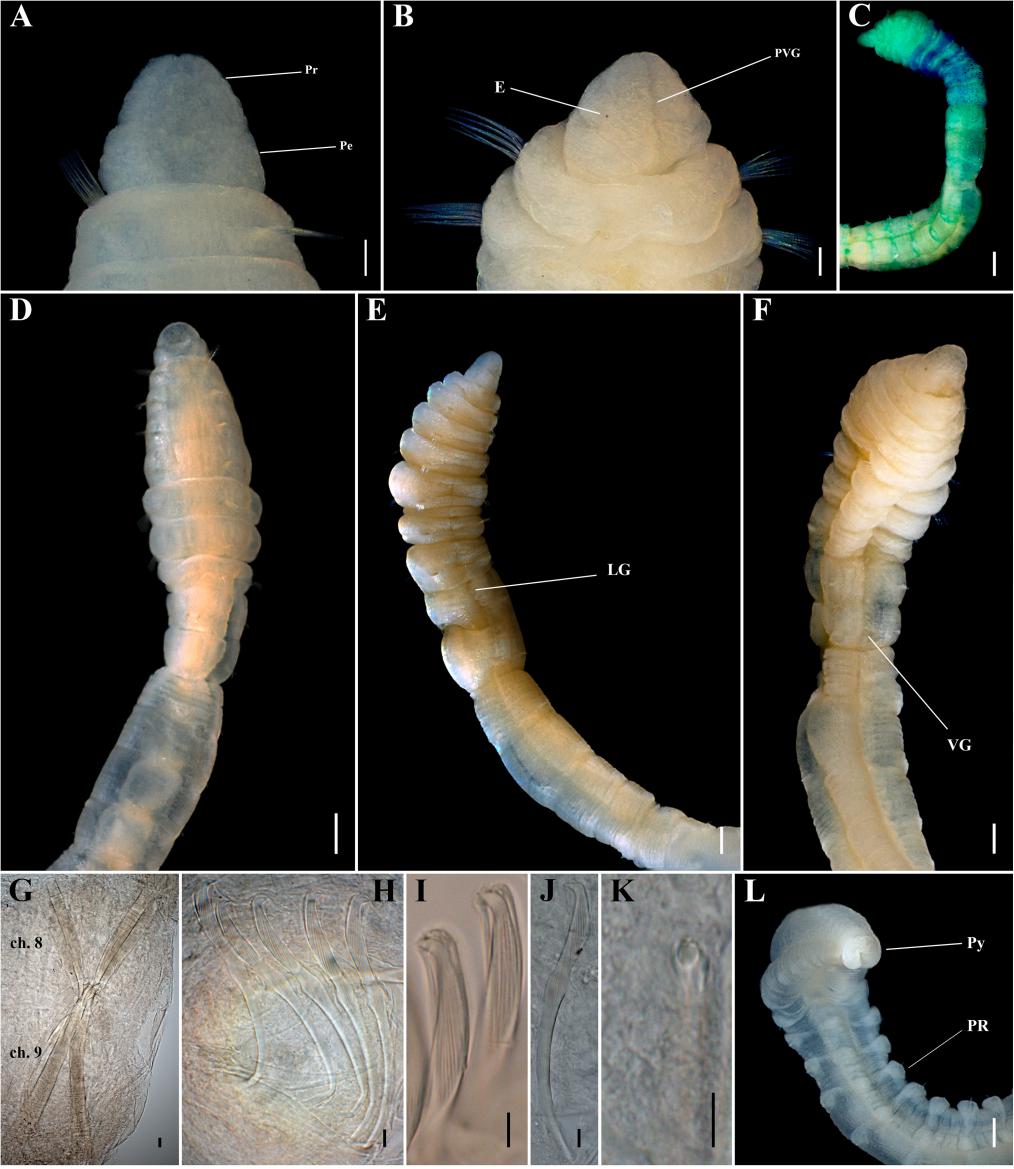
*Capitella nonatoi* sp. n. (A) Anterior end, dorsal view; (B) Anterior end, ventral view; (C) Methyl green staining pattern; (D) Thoracic region of a male specimen, dorsal view; (E) Thoracic region of a male specimen, lateral view; (F) Thoracic region of a male specimen, ventral view; (G) Genital hooks; (H) Thoracic hooded hook, lateral view; (I) Thoracic hooded hook, frontal view; (J) Abdominal hooded hook, lateral view; (K) Abdominal hooded hook, frontal view; (L) Posterior end and pygidium. Ch: chaetiger. E: eyespot. Pe: peristomium. Pr: prostomium. LG: lateral groove. VG: ventral groove. PVG: prostomial ventral groove. PR: parapodial ridges. Scale bars: A, B, F, L, 0.1 mm; C, D, E, 0.2 mm; G, 20 μm; H, I, J, K, 5 μm.

**Fig 12:**
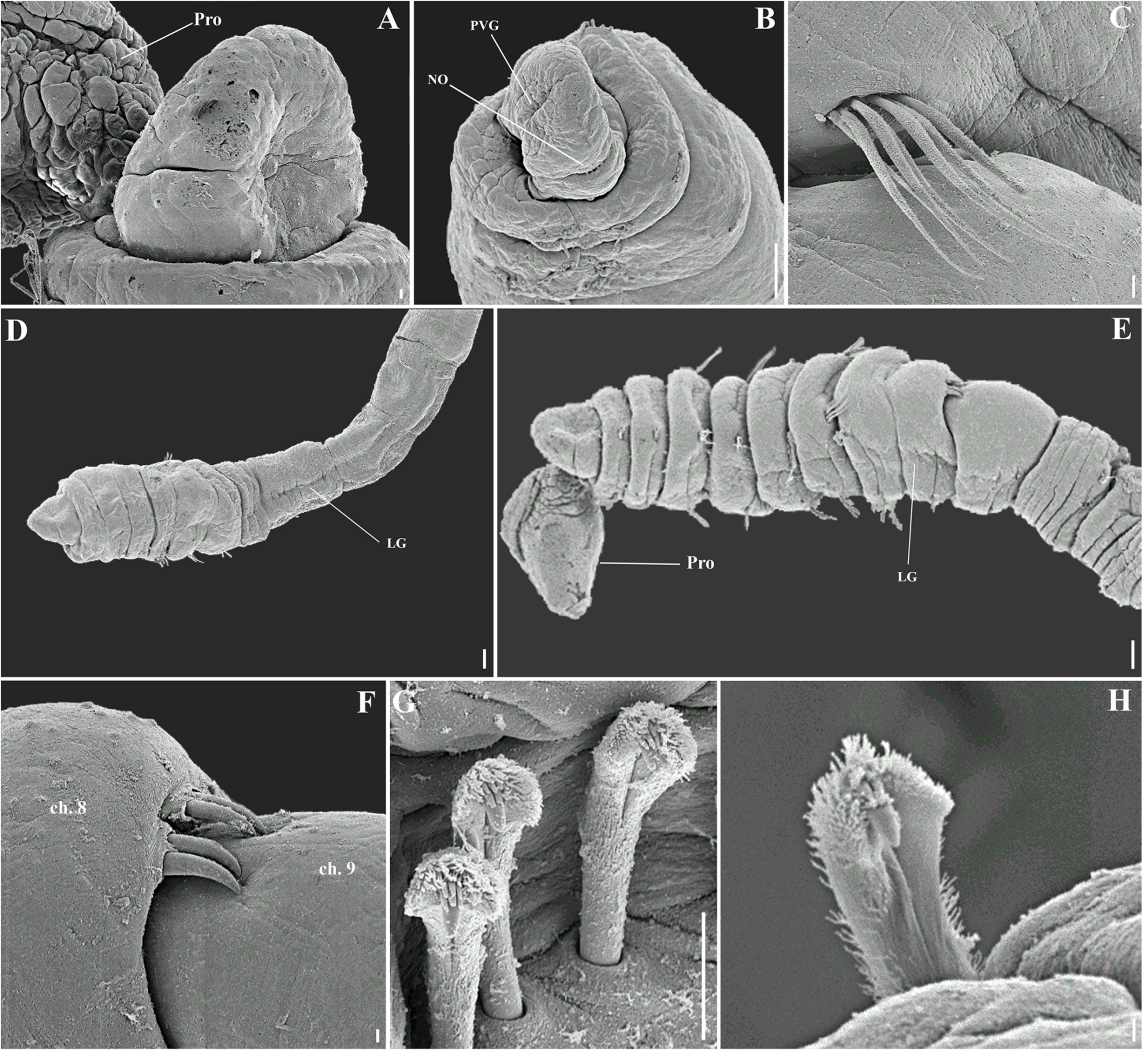
*Capitella nonatoi* sp. n., SEM. (A) Anterior end, dorso-lateral view; (B) Anterior end, ventro-lateral view; (C) Capillary chaetae; (D) Thoracic region of a female specimen, lateral view; (E) Thoracic region of a male specimen, lateral view; (F) Genital hooks; (G) Thoracic hooded hooks, frontal view; (H) Abdominal hooded hook, frontal view. Ch: chaetiger. LG: lateral groove. NO: nuchal organ. PVG: prostomial ventral groove. Pro: proboscis. Scale bars: A, C, F, 10 μm; B, D, E, 0.1 mm; G, H, 1 μm.

**Holotype:** São Paulo, Araçá Bay: ZUEC POL 17585: 23°48'39,4"S − 45°24'26,2"W; tidal flat; station 77(2); coll. 24 Jun 2013; 1 spec.

**Paratypes:** São Paulo, Araçá Bay: ZUEC POL 17460 –paratypes 1 − 3: 23°48'37,4"S − 45°24'21,4"W; tidal flat; station 80(1); coll. 24 Jun 2013; 3 specs. ZUEC POL 17461 –paratypes 4 − 8: 23°48'37,4"S − 45°24'21,4"W; tidal flat; station 6(1); coll. 13 Oct 2012; 5 specs. MNRJP 995 –paratypes 9 − 11: 23°48'51,4''S − 45°24'26,5''W; mangrove; station 124M; coll. 10 Jul 2014; 3 specs. MNRJP 996 –paratypes 12 − 15: 23°48'37,4"S − 45°24'21,4"W; tidal flat; station 80(4); coll. 24 Jun 2013; 4 specs.

**Additional material examined (S1 Appendix):** São Paulo, Araçá Bay (3,463 specs.); Rio de Janeiro, Itaipú Lagoon (15 specs.); Paraná, Paranaguá Bay (2 specs.); Pará, Caetê Bay (11 specs.).

**Description.** Based on type material, additional material and specimens examined by SEM. Size range of material examined (complete individuals) 3.1–15.0 mm long (holotype 7.5 mm), 0.3–0.7 mm wide (holotype 0.45 mm) and 31–71 chaetigers (holotype 49 chaetigers). Specimens widest anteriorly, gradual narrowing posteriorly. Color in alcohol yellowish. Prostomium short, rounded, as wide as long, with a dorsal smooth depression (Figs 10A; 11A and12A) and a ventral groove (Figs 10B; 11B and 12B). Peristomium forming an incomplete achaetous ring, conspicuous dorsal and laterally (Figs 10A and 10B; 11A and 11B), similar width than prostomium; eyespots present, a latero-ventral reddish spot (Figs 10B and 11B). Nuchal organ visible (Fig 12B). Chaetigers 1–5 gradually increasing in size, chaetiger 5 the largest; chaetigers 6–7 gradually decreasing in size; chaetigers 8 and 9 quadrangular in females and rounded in males; chaetigers 5–9 with mid-ventral and lateral groove (Figs 10C and 10D; 11D, 11E and 11F; 12D and 12E). Adult specimens with unilimbate capillaries (Fig 12C) in notopodia and neuropodia of chaetigers 1–7 and hooded hooks in notopodia and neuropodia of chaetigers 8–9. Notosetae arranged in a single irregular row of 3–15 capillaries and 5–8 hooded hooks; neurosetae arranged in a single irregular row of 4–17 capillaries and 5–8 hooded hooks. Thoracic hooded hooks with a pointed and large main fang at a right angle to the shaft, surmounted by 6 apical teeth arranged in two rows (3 basally and 3 in superior row); short anterior shaft; developed node; long and curved posterior shaft; short and smooth hood (Figs 10F and 10H; 11H and 11I and 12G). All chaetae emerging from the last third of the chaetiger. Chaetiger 8 with 4 straight external genital spines (2 fascicles), with tips sharply curved, narrower and shorter than those of chaetiger 9; chaetiger 9 with 4 straight embedded genital spines (2 fascicles), with tips sharply curved, wider and larger than those of chaetiger 8 (Figs 11G; 12E and 12F). Division between thorax and abdomen prominent. Abdominal chaetigers as long as wide; chaetigers with 2–5 hooded hooks in notopodia and 4–10 in neuropodia, reducing to 2 hooded hooks in notopodia and 4 in neuropodia. In far posterior chaetigers, neuropodial hooded hooks emerging from parapodial ridges (Figs 10J and 11L). Abdominal hooded hooks smaller than the thoracics, with a pointed main fang, upward curved, surmounted by three teeth arranged in two rows (2 basally and 1 on superior row); short shaft; developed node; long and curved posterior shaft; short and smooth hood (Figs 10G and 10I; 11J and 11K and 12H). Branchiae absent. Pygidium a small simple lobe without anal cirri (Figs 10J and 11L).

**Methyl green staining pattern.** Chaetigers 5–8 darkly stained, chaetigers 8 and 9 and the first two abdominal with dark speckles (Fig 11C).

**Biology.** All specimens without genital spines, with oocytes in the abdominal region confirming they are female specimens.

**Remarks.** *Capitella nonatoi* sp. n. belongs to a group of species of *Capitella* with capillary chaetae on chaetigers 1–7 and hooded hooks on chaetigers 8 and 9. *Capitella teleta* resembles *C*. *nonatoi* sp. n. in the overall body shape, peristomium forming an incomplete ring, a very conspicuous nuchal organ and presence of eyespots. However, the former differs from *C*. *nonatoi* sp. n. in having a flattened and long prostomium, while in *C*. *nonatoi* sp. n. the prostomium is short and rounded. The differences are also related to the number and features of the abdominal hooded hooks and number of genital spines. *Capitella teleta* has 4 − 6 hooks in notopodia and 5 − 6 in neuropodia, with six teeth above main fang, while *C*. *nonatoi* sp. n. has 2 − 5 hooded hooks in notopodia and 4 − 10 in neuropodia, with three teeth arranged in two rows. Furthermore, *C*. *teleta* has 6 − 8 genital spines on chaetiger 8 and *C*. *nonatoi* sp. n. has four. *Capitella caribaeorum*, *C*. *iatapiuna* and *C*. *singularis* differ from *C*. *nonatoi* sp. n. by having a peristomium forming a complete ring and the absence of eyespots. Besides *C*. *perarmata*, *C*. *capitata* and *C*. *dizonata* also have a peristomium forming an incomplete ring but they differ from *C*. *nonatoi* sp. n. by the absence of eyespots and features of the hooded hooks such as number of teeth and their distribution above main fang. *Capitella nonatoi* sp. n. can be distinguished by its short and rounded prostomium with a dorsal smooth depression and a ventral groove, and eyespots.

**Etymology.** This species was named *in memorian* of Professor Edmundo Ferraz Nonato, who was the pioneer on taxonomy of Polychaeta in Brazil and who had influenced many researchers with his dedication, knowledge and passion for polychaetes and science.

**Habitat.** Intertidal region, in fine sand and mangrove sediments.

**Type locality.** Aracá Bay, São Sebastião, São Paulo, Brazil (South Atlantic Ocean).

**Distribution.** South Atlantic Ocean: Brazil (states of Bahia, Pará, Paraná, Pernambuco, Rio de Janeiro and São Paulo).

### Molecular analysis

**Fig 13:**
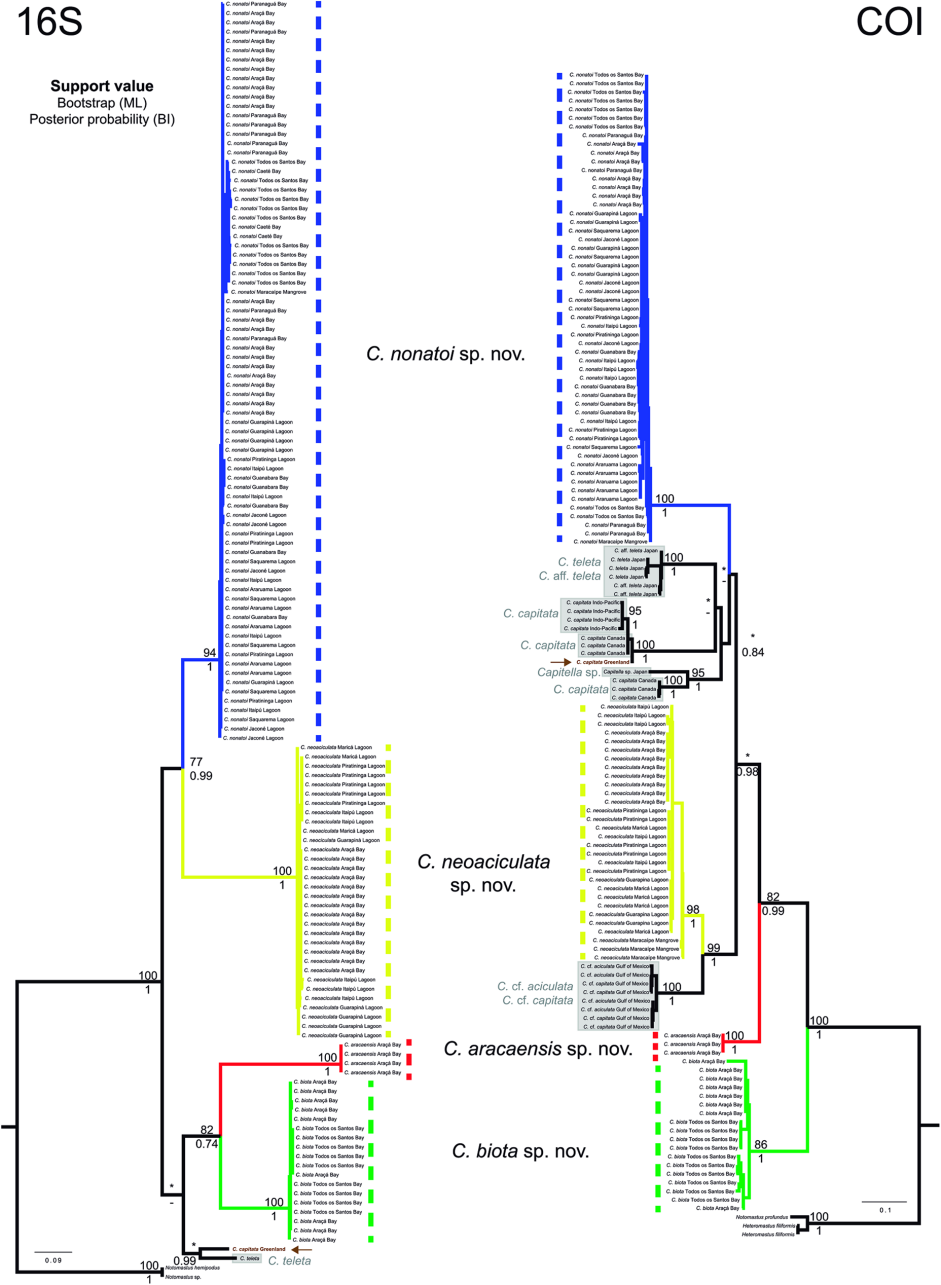
Phylogenetic trees based on maximum likelihood for 16S (left) and COI (right) genes. The numbers in nodes represent the support values for ML (bootstrap—on top) and BI (posterior probability—below). The BI trees were not represented. The scale bar represents the average nucleotide substitutions per site. Brown arrow shows the *Capitella capitata* from the type-locality (west Greenland). Gray boxes indicate Genbank sequences from *Capitella* species. Asterisks (*) indicates that the support value was lower than 70 (ML) or 0.7 (BI), and a dash (-) indicates that the branch was not recovered.

The phylogenetic analyses were based on 134 sequences of 16S and 106 of COI. The final alignment consisted of 558 bp for 16S and 655 bp for COI. Excluding the outgroup species, the 16S sequence had 215 (38.5%) variable sites, while the COI sequence had 273 (41.7%). The maximum likelihood and Bayesian inference method recovered the same species, as had been previously identified here by morphological characteristics (*C*. *aracaensis* sp. n., *C*. *biota* sp. n., *C*. *neoaciculata* sp. n. and *C*. *nonatoi* sp. n.). The four species clades were reciprocally monophyletic and were well supported in all datasets (COI, 16S and concatenated) and recovery methods (Fig 13 and S1 Fig). The *Capitella nonatoi* sp. n. clade included individuals from 12 sampling sites, while *C*. *neoaciculata* sp. n. was present in 6 sites, *C*. *biota* sp. n. in two sites and *C*. *aracaensis* sp. n. in only one site.

The mean pairwise genetic distance within species based on the K2P ranged from 0% (*C*. *aracaensis* sp. n.) to 0.5% (*C*. *neoaciculata* sp. n.) in 16S and from 0% (*C*. *aracaensis* sp. n.) to 2.3% (*C*. *biota* sp. n.) in COI (Table 2). In relation to the genetic distance among the *Capitella* species, the K2P values ranged from 24.4% (*C*. *nonatoi* sp. n. × *C*. *neoaciculata* sp. n.) to 46.4% (*C*. *aracaensis* sp. n. × *C*. *neoaciculata* sp. n.) in 16S and from 15.7% (*C*. *aracaensis* sp. n. × *C*. *neoaciculata* sp. n.) to 26.5% (*C*. *nonatoi* sp. n. × *C*. *capitata*) in COI (Table 2). As expected, the values based on *p*-distance were lower than the K2P (S1 Table).

**Table 2:** Intraspecific (in bold) and interspecific mean pairwise genetic distance based on Kimura-2-parameters (K2P) for 16S (on top) and COI (below). All values are in percentage. Number in parenthesis represents the standard error.

	1	2	3	4	5
1—*C*. *nonatoi* sp. n.	**0.4 (0.2)**				
	**1.6 (0.5)**				
2—*C*. *neoaciculata* sp. n.	25.4 (3.3)	**0.4 (0.2)**			
	21.5 (3.3)	**1.7 (0.5)**			
3—*C*. *biota* sp. n.	31.8 (3.7)	35.9 (4.1)	**0.4 (0.2)**		
	17.9 (2.9)	20.5 (3.2)	**2.2 (0.6)**		
4—*C*. *aracaensis* sp. n.	33.6 (4.0)	46.4 (5.3)	32.8 (3.8)	**0.0 (0.0)**	
	20.6 (3.2)	15.7 (2.7)	20.3 (3.2)	**0.0 (0.0)**	
5—*C*. *capitata*	26.0 (3.5)	29.6 (3.8)	31.7 (3.9)	35.7 (4.4)	**nd**
	26.5 (3.9)	19.0 (3.1)	22.9 (3.4)	21.2 (3.4)	**1.7 (0.7)**

## Discussion

The use of mtDNA sequences for species identification may speed up global diversity estimations [65], while the robustness of the morphological information improves the quantity and quality of species descriptions, as well as the understanding of the morphological adaptations in their evolutionary history [66–67]. Our morphological and molecular data were congruent and supported the existence of four different species of *Capitella*, all of them rather distinct from *Capitella capitata*, increasing the biodiversity of the genus along the Brazilian coast.

The delimitation of the boundaries of the various *Capitella* species along Brazilian waters has revealed a hidden local diversity and may enhance assessments of environmental health [68] as an indicator of organic pollution [69–72]. Sympatric occurrences were observed in six sites; most species distributed along the coast, while one only at only one site (*Capitella aracaensis* sp. n.). Combined morphological and molecular approaches were also conducted and have yielded similar results [25–26, 73–74]. Indeed, these authors identified distinct species among the species complexes, increasing the richness of the target genus [25, 26, 73, 74]. Interestingly, the taxonomic confusion surrounding *Capitella* species can be illustrated through a number of studies that focused on the genus, but were unable to identify specimens at the species level [75–79].

Our results indicate that many studies may have overlooked the richness of *Capitella* species due to the challenges in the morphological taxonomic identification, despite the ecological importance of the genus [7]. The four new species described here were morphologically differentiated from all congeners based mainly on the shape of the prostomium, peristomium and thorax, as well as the shape and number of thoracic and abdominal hooded hooks, the shape of the pygidium and of the genital hooks. The differences of these characteristics among the *Capitella* species are summarized in a table in [5]. Although the morphology of the prostomium and peristomium may be affected by distinct fixation protocols [80], they were very informative characteristics and should be explored, as they are quite variable and diagnostic for species within the genus [3–6, 59]. We would like to highlight the fact that these new species were distinguished initially by a morphological analysis. The sequence data just confirmed our findings and helped to support the specific status of the recognized morphospecies.

Among these new species, the most widespread and abundant was *C*. *nonatoi* sp. n., with records in all sampled sites along 4,500 Km along the Brazilian coast, from the North (Pará) to the South (Paraná). Due to its high abundance and wide distribution, this species has likely been misidentified as *C*. *capitata* by previous studies [81–84]. The second most widespread species was *C*. *biota* sp. n., which was reported from the Northeast (Bahia) to the Southeast (São Paulo), ranging over an area of 1,630 Km. *Capitella aracaensis* sp. n. was more rare in our sampling, with low abundances and was restricted to one site of the Southeast Brazil (São Paulo). We suggested that this species is rare because Araçá Bay [64], the type locality, was exhaustively and systematically sampled over a large area during the four years of the BIOTA-FAPESP Program (Thematic Grants, Process 2011/50317-5). During these four years we found just a few specimens of *Capitella aracaensis* sp. n., meanwhile the other three species were frequent and abundant at the same site. The discrepancies regarding the geographic distributions of these species could be explained by sampling effort, biological aspects such as their differing reproduction strategies [85], larvae development, colonization capacity [86], and physiological tolerances [17].

The low levels of intraspecific molecular variations recorded for all the four *Capitella* species (with a maximum of 2.2% for *C*. *biota* sp. n.) were expected within the same lineage. Usually values lower than 6% indicate that lineages are not composed of some sub-complex of the species [74]. On the other hand, high interspecific variation, as was observed here, 15.7% up to 26.5% for COI, and 24.4% up to 46.4% for 16S was observed in other congeners, including between the deep-sea species, for example *C*. *iatapiuna* and *C*. *teleta* (20% for 16S) [5]. A similar molecular interspecific variation was found for errant [24, 87], and sedentary annelids [27]. The COI values were compatible with the 10-fold rule of species delimitation [88], supporting the hypothesis that the sample groups indeed represent four different species. Therefore, no less conservative method of species delimitation based on cluster recognition [89] was necessary to confirm this status. Recently, COI sequences have been applied to clarify the systematics of the *Capitella capitata* complex [90–91]. A similar result was found in the Gulf of Mexico, with two species under the name *C*. *capitata*, with 21.7% of K2P divergence [90], while [91] found the same haplotype of *C*. *teleta* both on the Atlantic coast of USA and Japan. Despite the fact that these two studies are inconclusive regarding their morphological approaches, intermediate morphologies between the species [90] and an intraspecific variability within the species [91] were found.

Thus, considering the intra and inter-specific variation calculated for these particular mitochondrial genes, we concluded that these divergences support the distinction among the four morphologically delimitated species. Furthermore, a genetic distance comparison showed that the COI values were higher than 16S values when considering intraspecific comparisons. However, in relation to the differences between species, the COI values were smaller than the 16S values.

In addition to morphological and molecular studies confirming the cosmopolitan status of some species [92–93], our findings support restricting the geographical range of *C*. *capitata*, as was previously proposed by Blake [3]. Furthermore, we revealed a hidden diversity within this genus along the Brazilian coast. Despite the fragmentation of the *C*. *capitata* complex in at least four species, this does not indicate that each species has a restricted distribution, as frequently expected in this type of study. Thus, even for marine invertebrates with a supposedly low potential for dispersion, the population cohesion along a wide distribution can be maintained.

Finally, this study showed the importance of exhaustive and systematic sampling to unveil cryptic and rare species. Furthermore, this result emphasized the effectiveness of careful and accurate morphological study as a tool to differentiate species of *Capitella*.

## Supporting information

S1 Appendix Material examined. List of additional material examined of *Capitella* species.
(DOCX)

S1 Fig Phylogenetic tree based on maximum likelihood for concatenated dataset (16S + COI). The number in nodes represent the support values for ML (bootstrap–on top) and BI (posterior probability–below). The BI trees were not represented. The scale bar represents the average nucleotide substitutions per site. Brown arrow shows the *Capitella capitata* from type-locality (west Greenland). One asterisk (*) indicates that only COI sequence was used and two asterisks (**) indicate that only 16S sequence was used. A tilde (~) indicates that the support value was lower than 70 (ML) or 0.7 (BI), and a dash (-) indicates that the branch was not recovered.
(TIF)

S1 Table Genetic distance. Intraspecific (in bold) and interspecific mean pairwise genetic distances based on *p*-distance for 16S (on top) and COI (below). All values are in percentage. The number in parenthesis represents the standard error.
(DOCX)
