# Supplementary material for: Demystifying the Capitella capitata complex (Annelida, Capitellidae) diversity by morphological and molecular data along the Brazilian coast
Source: PLoS One. 2017 May 31;12(5):e0177760. doi: 10.1371/journal.pone.0177760 (PMC5451021; doi:10.1371/journal.pone.0177760)
Supplement: S1 Appendix — List of additional material examined of Capitella species. (DOCX) [file pone.0177760.s001.docx]

APPENDIX

MATERIAL EXAMINED

***Capitella aracaensis* sp. n. Silva & Amaral**

**Additional material examined. São Paulo, Araçá Bay:** ZUEC POL 17437: 23º48'57,2"S − 45º24'29,3"W; tidal flat; station 75(3)A; coll. 09 May 2012; 1 spec. ZUEC POL 17438: 23º48'55,2"S − 45º24'30,2"W; tidal flat; station 111(1)A; coll. 30 Jul 2012; 1 spec. ZUEC POL 17439: 23º48'50,2"S − 45º24'28,2"W; tidal flat; station 8(3); coll. 08 Oct 2012; 1 spec. ZUEC POL 17440: 23º48'50,7"S − 45º24'28,0"W; tidal flat; station 51(3)A; coll. 24 Feb 2012; 1 spec. ZUEC POL 17441: 23º48'52,9"S − 45º24'30,2"W; tidal flat; station 116(1)A; coll. 30 Jul 2012; 1 spec. ZUEC POL 17442: 23º48'45,5"S − 45º24'11,0"W; 0.5 m deep; station 13 Mc(4); coll. 05 Jun 2014; 1 spec. ZUEC POL 17443: 23º48'51,4''S − 45º24'26,5''W; mangrove; station 63M; coll. 19 Mar 2014; 1 spec. ZUEC POL 17444: 23º48'45,5"S − 45º24'11,0"W; 0.5 m deep; station 13 Mc(2); coll. 05 Jun 2014; 1 spec. ZUEC POL 17445: 23º48'44,4"S − 45º24'22,3"W; tidal flat; station 153(1)G; coll. 24 Feb 2013; 1 spec. ZUEC POL 17446: 23º48'53,4"S − 45º24'28,7"W; tidal flat; station 81(3)A; coll. 09 May 2012; 1 spec. ZUEC POL 17447: 23º48'51,4''S − 45º24'26,5''W; mangrove; station 65M; coll. 19 Mar 2014; 2 specs. ZUEC POL 17448: 23º48'37,4"S − 45º24'19,2"W; tidal flat; station 67(1); coll. 08 Feb 2012; 5 specs. ZUEC POL 17449: 23º48'51,4''S − 45º24'26,5''W; mangrove; station 58M; coll. 19 Mar 2014; 1 spec. ZUEC POL 17450: 23º48'37,4"S − 45º24'19,2"W; tidal flat; station 67(1); coll. 08 Feb 2012; 2 specs. ZUEC POL 17452: 23º48'36,1"S − 45º24'19,5"W; tidal flat; station 136(3)A; coll. 01 Aug 2012; 1 spec. ZUEC POL 17453: 23º48'47,5"S − 45º24'25,1"W; tidal flat; station 22(1)A; coll. 29 Sep 2011; 1 spec. ZUEC POL 17454: 23º48'39,2"S − 45º24'17,5"W; tidal flat; station 48(4); coll. 24 Feb 2013; 1 spec. ZUEC POL 17455: 23º48'43,9"S − 45º24'16,5"W; tidal flat; station 31(1)A; coll. 30 Sep 2011; 1 spec. ZUEC POL 17456: 23º48'36,1"S − 45º24'19,5"W; tidal flat; station 68(3); coll. 08 Feb 2012; 1 spec. ZUEC POL 17458: 23º48'46,6''S − 45º24'29,8''W; mangrove; station 25M; coll. 18 Mar 2014; 1 spec.

***Capitella biota* sp. n. Silva & Amaral**

**Additional material examined. São Paulo, Araçá Bay:** ZUEC POL 16733: 23º48'51,4''S − 45º24'26,5''W; mangrove; station 55M; coll. 19 Mar 2014; 6 specs. ZUEC POL 16734: 23º48'51,4''S − 45º24'26,5''W; mangrove; station 70M; coll. 19 Mar 2014; 1 spec. ZUEC POL 16735: 23º48'51,4''S − 45º24'26,5''W; mangrove; station 70M; coll. 19 Mar 2014; 1 spec. ZUEC POL 16736: 23º48'46,6''S − 45º24'29,8''W; mangrove; station 39M; coll. 19 Mar 2014; 2 specs. ZUEC POL 16737: 23º48'51,4''S − 45º24'26,5''W; mangrove; station 62M; coll. 19 Mar 2014; 1 spec. ZUEC POL 16738: 23º48'51,4''S − 45º24'26,5''W; mangrove; station 143M; coll. 10 Jul 2014; 1 spec. ZUEC POL 16739: 23º48'46,6''S − 45º24'29,8''W; mangrove; station 119M; coll. 10 Jul 2014; 2 specs. ZUEC POL 16740: 23º48'51,4''S − 45º24'26,5''W; mangrove; station 63M; coll. 19 Mar 2014; 5 specs. ZUEC POL 16741: 23º48'51,4''S − 45º24'26,5''W; mangrove; station 63M; coll. 19 Mar 2014; 1 spec. ZUEC POL 16742: 23º49' 01,7"S − 45º24'24,9"W; tidal flat; station 37(1)B; coll. 05 Feb 2012; 2 specs. ZUEC POL 16743: 23º49' 01,7"S − 45º24'24,9"W; tidal flat; station 37(2)A; coll. 05 Feb 2012; 14 specs. ZUEC POL 16744: 23º48'51,4''S − 45º24'26,5''W; mangrove; station 64M; coll. 19 Mar 2014; 9 specs. ZUEC POL 16746: 23º49' 02,4"S − 45º24'23,0 "W; tidal flat; station 70(1)A; coll. 09 May 2012; 1 spec. ZUEC POL 16747: 23º49' 01,7"S − 45º24'24,9"W; tidal flat; station 37(1)A; coll. 05 Feb 2012; 3 specs. ZUEC POL 16748: 23º48'57,5"S − 45º24'29,6"W; tidal flat; station 76(1)A; coll. 09 May 2012; 1 spec. ZUEC POL 16749: 23º49'01,7"S − 45º24'24,9"W; tidal flat; station 148(3)G; coll. 25 Feb 2013; 2 specs. ZUEC POL 16750: 23º48'51,4''S − 45º24'26,5''W; mangrove; station 127M; coll. 10 Jul 2014; 4 specs. ZUEC POL 16751: 23º48'51,4''S − 45º24'26,5''W; mangrove; station 135M; coll. 10 Jul 2014; 1 spec. ZUEC POL 16752: 23º48'37,4"S − 45º24'21,4"W; tidal flat; station 117(1); coll. 17 Sep 2013; 1 spec. ZUEC POL 16753: 23º48'51,4''S − 45º24'26,5''W; mangrove; station 144M; coll. 10 Jul 2014; 2 specs. ZUEC POL 16754: 23º48'46,6''S − 45º24'29,8''W; mangrove; station 102M; coll. 10 Jul 2014; 3 specs. ZUEC POL 16755: 23º48'37,4"S − 45º24'21,4"W; tidal flat; station 117(2); coll. 17 Sep 2013; 1 spec. ZUEC POL 16756: 23º48'39,4"S − 45º24'26,2"W; tidal flat; station 77(3); coll. 24 Jun 2013; 1 spec. ZUEC POL 16757: 23º48'37,4"S − 45º24'21,4"W; tidal flat; station 80(1); coll. 24 Jun 2013; 1 spec. ZUEC POL 16758: 23º48'37,4"S − 45º24'21,4"W; tidal flat; station 100(2)Port; coll. 25 Feb 2013; 1 spec. ZUEC POL 16759: 23º48'40,1"S − 45º24'23,1"W; tidal flat; station 116(2); coll. 17 Sep 2013; 2 specs. ZUEC POL 16760: 23º48'37,4"S − 45º24'21,4"W; tidal flat; station 117(4); coll. 17 Sep 2013; 1 spec. ZUEC POL 16761: 23º48'39,4"S − 45º24'26,2"W; tidal flat; station 114(1); coll. 17 Sep 2013; 2 specs. ZUEC POL 16762: 23º48'58,3"S − 45º24'25,4"W; tidal flat; station 149(1)G; coll. 25 Feb 2013; 1 spec. ZUEC POL 16764: 23º48'54,4''S − 45º24'26,5''W; mangrove; station 7M; coll. 17 Mar 2014; 2 specs. ZUEC POL 16765: 23º48'46,6''S − 45º24'29,8''W; mangrove; station 103M; coll. 10 Jul 2014; 4 specs. ZUEC POL 16766: 23º48'54,4''S − 45º24'26,5''W; mangrove; station 96M; coll. 10 Jul 2014; 1 spec. ZUEC POL 16767: 23º48'01,7"S − 45º24'24,9"W; tidal flat; station 138(3)G; coll. 10 Oct 2012; 4 specs. ZUEC POL 16768: 23º48'39,4"S − 45º24'26,2"W; tidal flat; station 77(2); coll. 24 Jun 2013; 1 spec. ZUEC POL 16769: 23º48'45,8"S − 45º24'28,9"W; tidal flat; station 152(2)G; coll. 23 Feb 2013; 4 specs. ZUEC POL 16770: 23º48'37,4"S − 45º24'21,4"W; tidal flat; station 100(2)Port; coll. 25 Feb 2013; 9 specs. ZUEC POL 16771: 23º48'45,8"S − 45º24'28,9"W; tidal flat; station 171(2)G; coll. 16 Sep 2013; 1 spec. ZUEC POL 16772: 23º48'45,8"S − 45º24'28,9"W; tidal flat; station 171(3)G; coll. 16 Sep 2013; 35 specs. ZUEC POL 16773: 23º48'51,4''S − 45º24'26,5''W; mangrove; station 69M; coll. 19 Mar 2014; 1 spec. ZUEC POL 16774: 23º48'54,4''S − 45º24'26,5''W; mangrove; station 78M; coll. 10 Jul 2014; 1 spec. ZUEC POL 16775: 23º48'51,4''S − 45º24'26,5''W; mangrove; station 126M; coll. 10 Jul 2014; 1 spec. ZUEC POL 16776: 23º48'51,4''S − 45º24'26,5''W; mangrove; station 135M; coll. 10 Jul 2014; 1 spec. ZUEC POL 16777: 23º48'54,4''S − 45º24'26,5''W; mangrove; station 82M; coll. 10 Jul 2014; 1 spec. ZUEC POL 16778: 23º48'51,4''S − 45º24'26,5''W; mangrove; station 134M; coll. 10 Jul 2014; 5 specs.

***Capitella neoaciculata* sp. n. Silva & Seixas**

**Additional material examined. São Paulo, Araçá Bay:** ZUEC POL 16786: 23º48'46,6''S − 45º24'29,8''W; mangrove; station 43M; coll. 18 Mar 2014; 2 specs. ZUEC POL 16787: 23º48'51,4''S − 45º24'26,5''W; mangrove; station 58M; coll. 19 Mar 2014; 9 specs. ZUEC POL 16788: 23º48'51,4''S − 45º24'26,5''W; mangrove; station 53M; coll. 19 Mar 2014; 3 specs. ZUEC POL 16789: 23º48'52,6"S − 45º24'31,3"W; tidal flat; station 83(1)A; coll. 07 May 2012; 1 spec. ZUEC POL 16790: 23º48'36,1"S − 45º24'19,5"W; tidal flat; station 34(3)A; coll. 29 Sep 2011; 1 spec. ZUEC POL 16791: 23º48'51,4''S − 45º24'26,5''W; mangrove; station 53M; coll. 19 Mar 2014; 21 specs. ZUEC POL 16792: 23º48'51,4''S − 45º24'26,5''W; mangrove; station 51M; coll. 19 Mar 2014; 16 specs. ZUEC POL 16793: 23º48'51,4''S − 45º24'26,5''W; mangrove; station 53M; coll. 18 Mar 2014; 2 specs. ZUEC POL 16794: 23º48'46,6''S − 45º24'29,8''W; mangrove; station 44M; coll. 18 Mar 2014; 3 specs. ZUEC POL 16795: 23º48'51,4''S − 45º24'26,5''W; mangrove; station 55M; coll. 19 Mar 2014; 9 specs. ZUEC POL 16796: 23º48'37,4"S − 45º24'21,4"W; tidal flat; station 117(1); coll. 17 Sep 2013; 13 specs. ZUEC POL 16797: 23º48'46,6''S − 45º24'29,8''W; mangrove; station 39M; coll. 19 Mar 2014; 1 spec. ZUEC POL 16798: 23º48'51,4''S − 45º24'26,5''W; mangrove; station 63M; coll. 19 Mar 2014; 1 spec. ZUEC POL 16799: 23º48'51,4''S − 45º24'26,5''W; mangrove; station 68M; coll. 19 Mar 2014; 17 specs. ZUEC POL 16800: 23º48'45,8"S − 45º24'28,9"W; tidal flat; station 162(1)G; coll. 24 Jun 2013; 1 spec. ZUEC POL 16801: 23º48'51,4''S − 45º24'26,5''W; mangrove; station 64M; coll. 19 Mar 2014; 8 specs. ZUEC POL 16802: 23º48'46,6''S − 45º24'29,8''W; mangrove; station 101M; coll. 10 Jul 2014; 2 specs. ZUEC POL 16803: 23º48'51,4''S − 45º24'26,5''W; mangrove; station 49M; coll. 19 Mar 2014; 1 spec. ZUEC POL 16804: 23º48'46,6''S − 45º24'29,8''W; mangrove; station 97M; coll. 10 Jul 2014; 1 spec. ZUEC POL 16805: 23º48'51,4''S − 45º24'26,5''W; mangrove; station 60M; coll. 19 Mar 2014; 13 specs. ZUEC POL 16806: 23º48'51,4''S − 45º24'26,5''W; mangrove; station 59M; coll. 19 Mar 2014; 19 specs. ZUEC POL 16807: 23º48'46,6''S − 45º24'29,8''W; mangrove; station 25M; coll. 18 Mar 2014; 12 specs. ZUEC POL 16808: 23º48'37,4"S − 45º24'21,4"W; tidal flat; station 117(2); coll. 17 Sep 2013; 34 specs. ZUEC POL 16809: 23º48'46,6''S − 45º24'29,8''W; mangrove; station 47M; coll. 18 Mar 2014; 1 spec. ZUEC POL 16810: 23º48'46,6''S − 45º24'29,8''W; mangrove; station 36M; coll. 18 Mar 2014; 20 specs. ZUEC POL 16811: 23º48'36,1"S − 45º24'19,5"W; tidal flat; station 68(3); coll. 08 Feb 2012; 6 specs. ZUEC POL 16812: 23º48'39,4"S − 45º24'26,2" W; tidal flat; station 114(2); coll. 17 Sep 2013; 1 spec. ZUEC POL 16813: 23º48'46,6''S − 45º24'29,8''W; mangrove; station 42M; coll. 19 Mar 2014; 3 specs. ZUEC POL 16814: 23º48'51,4''S − 45º24'26,5''W; mangrove; station 67M; coll. 19 Mar 2014; 18 specs. ZUEC POL 16815: 23º48'51,4''S − 45º24'26,5''W; mangrove; station 50M; coll. 19 Mar 2014; 22 specs. ZUEC POL 16817: 23º48'36,1"S − 45º24'19,5"W; tidal flat; station 68(2); coll. 08 Feb 2012; 7 specs. ZUEC POL 16818: 23º48'45,8"S − 45º24'28,9"W; tidal flat; station 172(1)G; coll. 16 Sep 2013; 1 spec. ZUEC POL 16819: 23º49'05,1"S − 45º24'19,9"W; tidal flat; station 91(1)G; coll. 24 Jun 2013; 1 spec. ZUEC POL 16820: 23º48'46,6''S − 45º24'29,8''W; mangrove; station 115M; coll. 10 Jul 2014; 1 spec. ZUEC POL 16821: 23º48'39,4"S − 45º24'26,2"W; tidal flat; station 77(3); coll. 24 Jun 2013; 1 spec. ZUEC POL 16822: 23º48'55,3"S − 45º24'31,5"W; tidal flat; station 7(2); coll. 12 Oct 2012; 7 specs. ZUEC POL 16823: 23º48'45,8"S − 45º24'28,9"W; tidal flat; station 172(1)G; coll. 16 Sep 2013; 6 specs. ZUEC POL 16824: 23º48'40,1"S − 45º24'23,1"W; tidal flat; station 79(4); coll. 26 Jun 2013; 2 specs. ZUEC POL 16825: 23º48'44,0"S − 45º24'29,9"W; tidal flat; station 39(3); coll. 23 Feb 2013; 3 specs. ZUEC POL 17373: 23º48'51,4''S − 45º24'26,5''W; mangrove; station 135M; coll. 10 Jul 2014; 6 specs. ZUEC POL 17374: 23º48'39,4"S − 45º24'26,2"W; tidal flat; station 3(4); coll. 14 Oct 2012; 1 spec. ZUEC POL 17375: 23º48'54,4''S − 45º24'26,5''W; mangrove; station 77M; coll. 10 Jul 2014; 2 specs. ZUEC POL 17376: 23º48'55,3"S − 45º24'31,5"W; tidal flat; station 7(4); coll. 12 Oct 2012; 2 specs. ZUEC POL 17377: 23º48'51,4''S − 45º24'26,5''W; mangrove; station 113M; coll. 10 Jul 2014; 2 specs. ZUEC POL 17378: 23º48'37,4"S − 45º24'21,4"W; tidal flat; station 117(2); coll. 17 Sep 2013; 1 spec. ZUEC POL 17379: 23º48'46,6''S − 45º24'29,8''W; mangrove; station 108M; coll. 10 Jul 2014; 2 specs. ZUEC POL 17380: 23º48'51,4''S − 45º24'26,5''W; mangrove; station 125M; coll. 10 Jul 2014; 3 specs. ZUEC POL 17381: 23º48'51,4''S − 45º24'26,5''W; mangrove; station 131M; coll. 10 Jul 2014; 4 specs. ZUEC POL 17382: 23º48'51,4''S − 45º24'26,5''W; mangrove; station 140M; coll. 10 Jul 2014; 16 specs. ZUEC POL 17383: 23º48'51,4''S − 45º24'26,5''W; mangrove; station 139M; coll. 10 Jul 2014; 17 specs. ZUEC POL 17385: 23º48'54,4''S − 45º24'26,5''W; mangrove; station 89M; coll. 10 Jul 2014; 1 spec. ZUEC POL 17386: 23º48'51,4''S − 45º24'26,5''W; mangrove; station 126M; coll. 10 Jul 2014; 2 specs. ZUEC POL 17387: 23º48'37,4"S − 45º24'21,4"W; tidal flat; station 80(3); coll. 24 Jun 2013; 2 specs. ZUEC POL 17388: 23º48'42,8"S − 45º24'24,0"W; tidal flat; station 174(2)G; coll. 17 Sep 2013; 1 spec. ZUEC POL 17389: 23º48'37,4"S − 45º24'21,4"W; tidal flat; station 117(4); coll. 17 Sep 2013; 68 specs. ZUEC POL 17390: 23º48'37,4"S − 45º24'21,4"W; tidal flat; station 116(3); coll. 17 Sep 2013; 2 specs. ZUEC POL 17391: 23º48'39,4"S − 45º24'26,2"W; tidal flat; station 114(1); coll. 17 Sep 2013; 2 specs. ZUEC POL 17392: 23º48'37,4"S − 45º24'21,4"W; tidal flat; station 80(1); coll. 24 Jun 2013; 1 spec. ZUEC POL 17393: 23º48'37,4"S − 45º24'21,4"W; tidal flat; station 80(2); coll. 24 Jun 2013; 3 specs. ZUEC POL 17394: 23º48'37,4"S − 45º24'21,4"W; tidal flat; station 80(4); coll. 24 Jun 2013; 6 specs. ZUEC POL 17395: 23º48'39,4"S − 45º24'26,2"W; tidal flat; station 114(3); coll. 17 Sep 2013; 2 specs. ZUEC POL 17396: 23º48'51,4''S − 45º24'26,5''W; mangrove; station 133M; coll. 10 Jul 2014; 9 specs. ZUEC POL 17397: 23º48'51,4''S − 45º24'26,5''W; mangrove; station 121M; coll. 10 Jul 2014; 2 specs. ZUEC POL 17398: 23º48'51,4''S − 45º24'26,5''W; mangrove; station 63M; coll. 19 Mar 2014; 2 specs. ZUEC POL 17400: 23º48'46,6''S − 45º24'29,8''W; mangrove; station 35M; coll. 19 Mar 2014; 8 specs. ZUEC POL 17401: 23º48'46,6''S − 45º24'29,8''W; mangrove; station 46M; coll. 18 Mar 2014; 1 spec. ZUEC POL 17402: 23º48'55,3"S − 45º24'31,5"W; tidal flat; station 1(4); coll. 12 Oct 2012; 2 specs. ZUEC POL 17403: 23º48'47,3"S − 45º24'31,4"W; tidal flat; station 7(1); coll. 12 Oct 2012; 5 specs. ZUEC POL 17404: 23º48'39,4"S − 45º24'26,2"W; tidal flat; station 40(2); coll. 24 Feb 2013; 1 spec. ZUEC POL 17405: 23º48'37,4"S − 45º24'21,4"W; tidal flat; station 117(3); coll. 17 Sep 2013; 3 specs. ZUEC POL 17406: 23º48'44,0"S − 45º24'29,9"W; tidal flat; station 39(4); coll. 23 Feb 2013; 1 spec. ZUEC POL 17407: 23º48'44,0"S − 45º24'29,9"W; tidal flat; station 2(4); coll. 12 Oct 2012; 1 spec. ZUEC POL 17408: 23º48'46,6''S − 45º24'29,8''W; mangrove; station 35M; coll. 19 Mar 2014; 10 specs. ZUEC POL 17409: 23º48'46,6''S − 45º24'29,8''W; mangrove; station 106M; coll. 09 Jul 2014; 1 spec. ZUEC POL 17410: 23º48'51,4''S − 45º24'26,5''W; mangrove; station 132M; coll. 10 Jul 2014; 8 specs. ZUEC POL 17411: 23º48'46,6''S − 45º24'29,8''W; mangrove; station 37M; coll. 18 Mar 2014; 4 specs. ZUEC POL 17412: 23º48'51,4''S − 45º24'26,5''W; mangrove; station 131M; coll. 10 Jul 2014; 1 spec. ZUEC POL 17413: 23º48'55,3"S − 45º24'31,5"W; tidal flat; station 81(1); coll. 25 Jun 2013; 1 spec. ZUEC POL 17414: 23º48'51,4''S − 45º24'26,5''W; mangrove; station 141M; coll. 10 Jul 2014; 3 specs. ZUEC POL 17415: 23º48'37,4"S − 45º24'21,4"W; tidal flat; station 117(3); coll. 17 Sep 2013; 30 specs. ZUEC POL 17416: 23º48'51,4''S − 45º24'26,5''W; mangrove; station 141M; coll. 10 Jul 2014; 11 specs. ZUEC POL 17417: 23º48'46,6''S − 45º24'29,8''W; mangrove; station 28M; coll. 18 Mar 2014; 16 specs. ZUEC POL 17418: 23º48'51,4''S − 45º24'26,5''W; mangrove; station 123M; coll. 10 Jul 2014; 2 specs. ZUEC POL 17419: 23º48'51,4''S − 45º24'26,5''W; mangrove; station 62M; coll. 19 Mar 2014; 1 spec. ZUEC POL 17420: 23º48'51,4''S − 45º24'26,5''W; mangrove; station 57M; coll. 19 Mar 2014; 1 spec. ZUEC POL 17421: 23º48'54,4''S − 45º24'26,5''W; mangrove; station 11M; coll. 17 Mar 2014; 1 spec. ZUEC POL 17422: 23º48'51,4''S − 45º24'26,5''W; mangrove; station 69M; coll. 19 Mar 2014; 5 specs. ZUEC POL 17423: 23º48'46,6''S − 45º24'29,8''W; mangrove; station 32M; coll. 18 Mar 2014; 17 specs. ZUEC POL 17424: 23º48'46,6''S − 45º24'29,8''W; mangrove; station 41M; coll. 18 Mar 2014; 4 specs. ZUEC POL 17425: 23º48'51,4''S − 45º24'26,5''W; mangrove; station 66M; coll. 19 Mar 2014; 8 specs. ZUEC POL 17426: 23º48'51,4''S − 45º24'26,5''W; mangrove; station 52M; coll. 19 Mar 2014; 1 spec. ZUEC POL 17427: 23º48'46,6''S − 45º24'29,8''W; mangrove; station 29M; coll. 18 Mar 2014; 2 specs. ZUEC POL 17428: 23º48'46,6''S − 45º24'29,8''W; mangrove; station 33M; coll. 18 Mar 2014; 6 specs. ZUEC POL 17429: 23º48'51,4''S − 45º24'26,5''W; mangrove; station 54M; coll. 18 Mar 2014; 1 spec. ZUEC POL 17430: 23º48'51,4''S − 45º24'26,5''W; mangrove; station 65M; coll. 18 Mar 2014; 6 specs. ZUEC POL 17431: 23º48'51,4''S − 45º24'26,5''W; mangrove; station 52M; coll. 18 Mar 2014; 11 specs. ZUEC POL 17432: 23º48'51,4''S − 45º24'26,5''W; mangrove; station 57M; coll. 18 Mar 2014; 3 specs. ZUEC POL 17433: 23º48'46,6''S − 45º24'29,8''W; mangrove; station 45M; coll. 18 Mar 2014; 1 spec. ZUEC POL 17434: 23º48'46,6''S − 45º24'29,8''W; mangrove; station 41M; coll. 18 Mar 2014; 2 specs. ZUEC POL 17435: 23º48'54,4''S − 45º24'26,5''W; mangrove; station 4M; coll. 17 Mar 2014; 3 specs. ZUEC POL 17436: 23º48'51,4''S − 45º24'26,5''W; mangrove; station 61M; coll. 19 Mar 2014; 14 specs. **Rio de Janeiro, Piratininga Lagoon:** ZUEC POL 17647: 22º57'1,39''S − 43º05'33,67''W; shallow subtidal; 0.2 m deep; coll. 13 Mar 2013; 34 specs. **Rio de Janeiro, Itaipu Lagoon:** ZUEC POL 17648: 22º57'57,86''S − 43º2'39,69''W; shallow subtidal; 0.5 m deep; coll. 06 Mar 2013; 45 specs.

***Capitella nonatoi* sp. n. Silva & Amaral**

**Additional material examined: São Paulo, Araçá Bay:** ZUEC POL 17459: 23º48'37,4"S − 45º24'21,4"W; tidal flat; station 80(1); coll. 24 Jun 2013; 4 specs. ZUEC POL 17462: 23º48'37,4"S − 45º24'21,4"W; tidal flat; station 6(1); coll. 13 Oct 2012; 1 spec. ZUEC POL 17463: 23º48'40,1"S − 45º24'23,1"W; tidal flat; station 79(4); coll. 24 Jun 2013; 9 specs. ZUEC POL 17464: 23º448'39,4"S − 45º424'26,2"W; tidal flat; station 77(2); coll. 24 Jun 2013; 1 spec. ZUEC POL 17465: 23º48'45,8"S − 45º24'28,9"W; tidal flat; station 172(1)G; coll. 16 Sep 2013; 3 specs. ZUEC POL 17466: 23º48'46,6''S − 45º24'29,8''W; mangrove; station 116M; coll. 08 Jul 2014; 35 specs. ZUEC POL 17467: 23º48'37,4"S − 45º24'21,4"W; tidal flat; station 80(4); coll. 24 Jun 2013; 1 spec. ZUEC POL 17468: 23º48'51,4''S − 45º24'26,5''W; mangrove; station 124M; coll. 10 Jul 2014; 27 specs. ZUEC POL 17469: 23º48'37,4"S − 45º24'21,4"W; tidal flat; station 80(1); coll. 24 Jun 2013; 37 specs. ZUEC POL 17470: 23º48'54,4''S − 45º24'26,5''W; mangrove; station 73M; coll. 10 Jul 2014; 39 specs. ZUEC POL 17471: 23º48'46,6''S − 45º24'29,8''W; mangrove; station 117M; coll. 09 Jul 2014; 27 specs. ZUEC POL 17472: 23º48'55,3"S − 45º24'31,5"W; tidal flat; station 44(4); coll. 25 Feb 2012; 72 specs. ZUEC POL 17473: 23º48'37,4"S − 45º24'21,4"W; tidal flat; station 80(4); coll. 24 Jun 2013; 76 specs. ZUEC POL 17581: 23º48'37,4"S − 45º24'21,4"W; tidal flat; station 80(2); coll. 24 Jun 2013; 83 specs. ZUEC POL 17582: 23º48'46,6''S − 45º24'29,8''W; tidal flat; station 119(3); coll. 16 Sep 2013; 4 specs. ZUEC POL 17583: 23º48'46,6''S − 45º24'29,8''W; tidal flat; station 117(1); coll. 17 Sep 2013; 28 specs. ZUEC POL 17584: 23º448'39,4"S − 45º424'26,2"W; tidal flat; station 77(2); coll. 24 Jun 2013; 88 specs. ZUEC POL 17586: 23º48'49,2"S − 45º24'22,5"W; tidal flat; station 171(2)G; coll. 16 Sep 2013; 31 specs. ZUEC POL 17587: 23º48'46,6''S − 45º24'29,8''W; tidal flat; station 117(4); coll. 17 Sep 2013; 48 specs. ZUEC POL 17588: 23º48'55,3"S − 45º24'31,5"W; tidal flat; station 7(2); coll. 12 Oct 2012; 142 specs. ZUEC POL 17589: 23º48'39,4"S − 45º24'26,2"W; tidal flat; station 3(2); coll. 12 Oct 2012; 124 specs. ZUEC POL 17590: 23º48'39,4''S − 45º24'26,2''W; tidal flat; station 114(4); coll. 17 Sep 2013; 82 specs. ZUEC POL 17591: 23º48'46,6''S − 45º24'29,8''W; mangrove; station 113M; coll. 09 Jul 2014; 36 specs. ZUEC POL 17592: 23º48'51,4''S − 45º24'26,5''W; mangrove; station 130M; coll. 10 Jul 2014; 19 specs. ZUEC POL 17593: 23º48'46,6''S − 45º24'29,8''W; mangrove; station 110M; coll. 08 Jul 2014; 1 spec. ZUEC POL 17594: 23º48'51,4''S − 45º24'26,5''W; mangrove; station 125M; coll. 10 Jul 2014; 63 specs. ZUEC POL 17595: 23º48'51,4''S − 45º24'26,5''W; mangrove; station 122M; coll. 10 Jul 2014; 36 specs. ZUEC POL 17596: 23º48'51,4''S − 45º24'26,5''W; mangrove; station 124M; coll. 10 Jul 2014; 45 specs. ZUEC POL 17597: 23º48'45,8"S − 45º24'28,9"W; tidal flat; station 172(3)G; coll. 17 Sep 2013; 81 specs. ZUEC POL 17598: 23º48'46,6''S − 45º24'29,8''W; tidal flat; station 117(2); coll. 17 Sep 2013; 94 specs. ZUEC POL 17599: 23º48'37,4"S − 45º24'21,4"W; tidal flat; station 6(3); coll. 13 Oct 2012; 63 specs. ZUEC POL 17600: 23º48'39,4"S − 45º24'26,2"W; tidal flat; station 3(4); coll. 12 Oct 2012; 84 specs. ZUEC POL 17601: 23º48'37,4"S − 45º24'21,4"W; tidal flat; station 6(2); coll. 13 Oct 2012; 98 specs. ZUEC POL 17602: 23º48'54,4''S − 45º24'26,5''W; mangrove; station 73M; coll. 10 Jul 2014; 4 specs. ZUEC POL 17603: 23º48'55,3"S − 45º24'31,5"W; tidal flat; station 7(1); coll. 12 Oct 2012; 161 specs. ZUEC POL 17604: 23º48'40,1"S − 45º24'23,1"W; tidal flat; station 5(2); coll. 12 Oct 2012; 1 spec. ZUEC POL 17605: 23º48'45,8"S − 45º24'28,9"W; tidal flat; station 142(1)G; coll. 14 Oct 2012; 55 specs. ZUEC POL 17606: 23º48'01,7"S − 45º24'24,9"W; tidal flat; station 138(2)G; coll. 14 Oct 2012; 16 specs. ZUEC POL 17607: 23º48'40,1"S − 45º24'24,9"W; tidal flat; station 116(3); coll. 17 Sep 2013; 130 specs. ZUEC POL 17608: 23º48'44,0"S − 45º24'29,9"W; tidal flat; station 39(4); coll. 23 Feb 2013; 34 specs. ZUEC POL 17609: 23º48'39,2"S − 45º24'17,5"W; tidal flat; station 48(1); coll. 24 Feb 2013; 2 specs. ZUEC POL 17610: 23º48'47,3"S − 45º24'31,4"W; tidal flat; station 1(4); coll. 12 Oct 2012; 8 specs. ZUEC POL 17611: 23º48'45,8"S − 45º24'28,9"W; tidal flat; station 142(2)G; coll. 14 Oct 2012; 119 specs. ZUEC POL 17612: 23º48'37,4"S − 45º24'21,4"W; tidal flat; station 6(1); coll. 13 Oct 2012; 63 specs. ZUEC POL 17613: 23º48'55,3"S − 45º24'31,5"W; tidal flat; station 7(3); coll. 12 Oct 2012; 130 specs. ZUEC POL 17614: 23º48'52,1"S − 45º24'23,5"W; tidal flat; station 13(1); coll. 14 Oct 2012; 1 spec. ZUEC POL 17615: 23º48'47,4"S − 45º24'13,9"W; tidal flat; station 20(3); coll. 12 Oct 2012; 1 spec. ZUEC POL 17616: 23º48'55,3"S − 45º24'31,5"W; tidal flat; station 7(4); coll. 12 Oct 2012; 152 specs. ZUEC POL 17617: 23º48'39,4"S − 45º24'26,2"W; tidal flat; station 3(1); coll. 12 Oct 2012; 43 specs. ZUEC POL 17618: 23º48'40,1"S − 45º24'23,1"W; tidal flat; station 5(3); coll. 14 Oct 2012; 2 specs. ZUEC POL 17619: 23º48'05,1"S − 45º24'19,9"W; tidal flat; station 17(4); coll. 13 Oct 2012; 1 spec. ZUEC POL 17620: 23º48'47,3"S − 45º24'31,4"W; tidal flat; station 1(1); coll. 12 Oct 2012; 1 spec. ZUEC POL 17621: 23º48'44,0"S − 45º24'29,9"W; tidal flat; station 2(4); coll. 12 Oct 2012; 1 spec. ZUEC POL 17622: 23º48'40,8"S − 45º24'14,0"W; tidal flat; station 146(1)G; coll. 13 Oct 2012; 6 specs. ZUEC POL 17623: 23º48'51,4"S − 45º24'26,5"W; mangrove; station 135M; coll. 10 Jul 2014; 30 specs. ZUEC POL 17624: 23º48'54,4"S − 45º24'26,5"W; mangrove; station 84M; coll. 10 Jul 2014; 34 specs. ZUEC POL 17625: 23º48'46,6''S − 45º24'29,8''W; tidal flat; station 117(4); coll. 17 Sep 2013; 1 spec. ZUEC POL 17626: 23º48'54,4"S − 45º24'26,5"W; mangrove; station 93M; coll. 10 Jul 2014; 19 specs. ZUEC POL 17627: 23º48'46,6"S − 45º24'29,8"W; mangrove; station 107M; coll. 09 Jul 2014; 31 specs. ZUEC POL 17628: 23º48'49,2"S − 45º24'22,5"W; tidal flat; station 171(2)G; coll. 16 Sep 2013; 1 spec. ZUEC POL 17629: 23º48'51,4"S − 45º24'26,5"W; mangrove; station 127M; coll. 10 Jul 2014; 35 specs. ZUEC POL 17630: 23º48'46,6"S − 45º24'29,8"W; mangrove; station 102M; coll. 09 Jul 2014; 29 specs. ZUEC POL 17631: 23º48'46,6"S − 45º24'29,8"W; mangrove; station 115M; coll. 09 Jul 2014; 22 specs. ZUEC POL 17632: 23º48'51,4"S − 45º24'26,5"W; mangrove; station 144M; coll. 09 Jul 2014; 18 specs. ZUEC POL 17633: 23º48'51,4"S − 45º24'26,5"W; mangrove; station 128M; coll. 09 Jul 2014; 11 specs. ZUEC POL 17634: 23º48'44,0"S − 45º24'29,9"W; tidal flat; station 2(3); coll. 12 Oct 2012; 1 spec. ZUEC POL 17635: 23º48'44,0"S − 45º24'29,9"W; tidal flat; station 2(2); coll. 12 Oct 2012; 1 spec. ZUEC POL 17636: 23º48'40,1"S − 45º24'23,1"W; tidal flat; station 5(1); coll. 14 Oct 2012; 1 spec. ZUEC POL 17637: 23º48'39,4"S − 45º24'26,2"W; tidal flat; station 3(3); coll. 14 Oct 2012; 9 specs. ZUEC POL 17638: 23º48'46,6"S − 45º24'29,8"W; mangrove; station 32M; coll. 18 Mar 2014; 19 specs. ZUEC POL 17639: 23º48'51,4"S − 45º24'26,5"W; mangrove; station 71M; coll. 19 Mar 2014; 17 specs. ZUEC POL 17640: 23º48'51,4"S − 45º24'26,5"W; mangrove; station 66M; coll. 19 Mar 2014; 146 specs. ZUEC POL 17641: 23º48'46,6"S − 45º24'29,8"W; mangrove; station 28M; coll. 18 Mar 2014; 13 specs. ZUEC POL 17642: 23º48'54,4"S − 45º24'26,5"W; mangrove; station 11M; coll. 18 Mar 2014; 33 specs. ZUEC POL 17643: 23º48'54,4"S − 45º24'26,5"W; mangrove; station 19M; coll. 17 Mar 2014; 58 specs. ZUEC POL 17644: 23º48'54,4"S − 45º24'26,5"W; mangrove; station 10M; coll. 17 Mar 2014; 19 specs. ZUEC POL 17645: 23º48'51,4"S − 45º24'26,5"W; mangrove; station 69M; coll. 17 Mar 2014; 121 specs. ZUEC POL 17646: 23º48'51,4"S − 45º24'26,5"W; mangrove; station 65M; coll. 17 Mar 2014; 120 specs. ZUEC POL 17649: 23º48'51,4"S − 45º24'26,5"W; mangrove; station 67M; coll. 19 Mar 2014; 168 specs. **Rio de Janeiro, Itaipu Lagoon:** ZUEC POL 17650: 22º57'57,86''S − 43º2'39,69''W; shallow subtidal; 0.5 m deep; coll. 13 Mar 2013; 15 specs. **Paraná, Paranaguá Bay:** ZUEC POL 17651: 25º30'44''S − 48º29'45''W; intertidal; station 1CS; coll. 09 Apr 2013; 2 specs. **Pará, Caete Bay:** ZUEC POL 17652: -0º55'S − 46º37'W; shallow subtidal; 0.5 m deep; coll. 28 Dec 2013; 11 specs.
