# Supplementary figures and images for: Demystifying the Capitella capitata complex (Annelida, Capitellidae) diversity by morphological and molecular data along the Brazilian coast
Source: PLoS One. 2017 May 31;12(5):e0177760. doi: 10.1371/journal.pone.0177760 (PMC5451021; doi:10.1371/journal.pone.0177760)

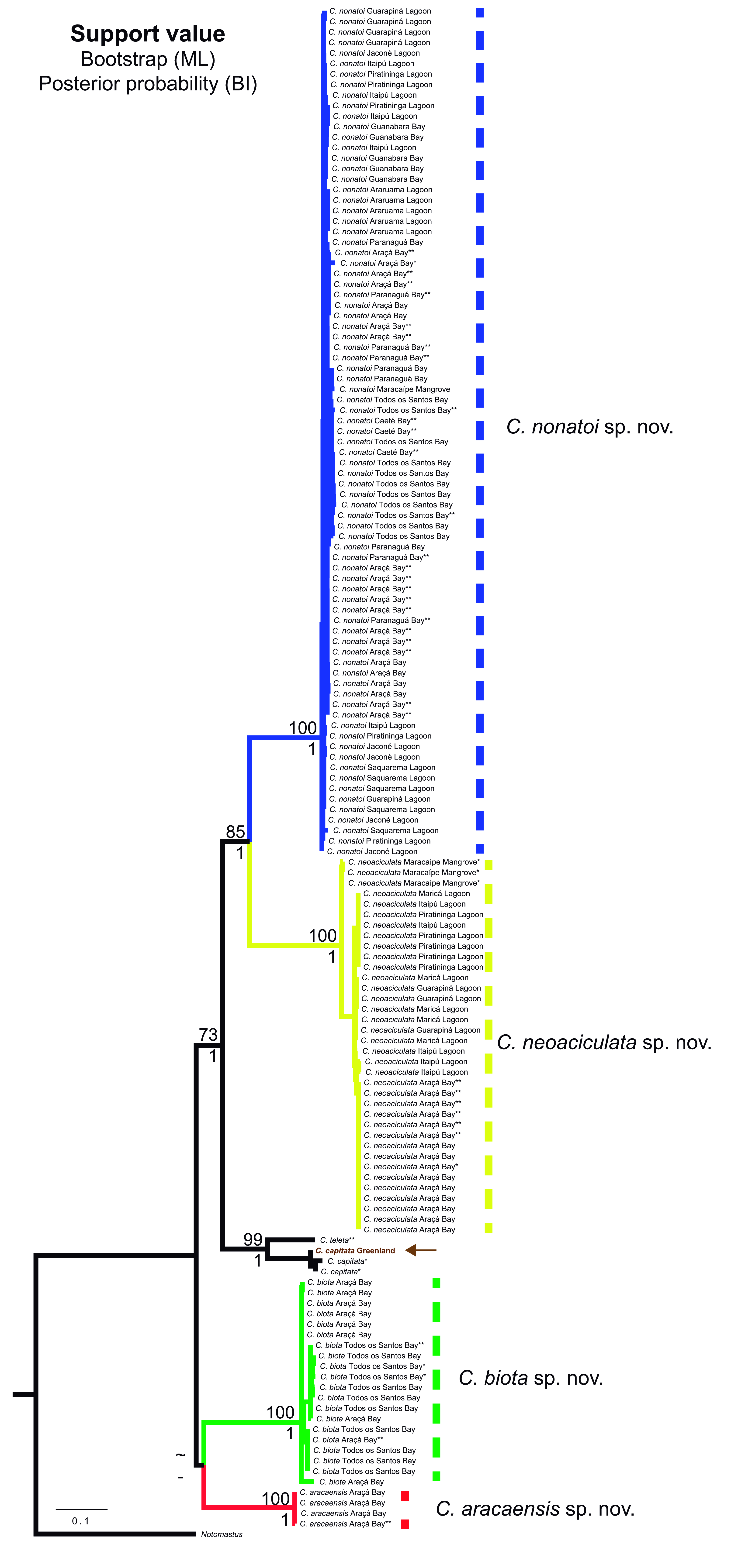

Supplement: S1 Fig — The number in nodes represent the support values for ML (bootstrap–on top) and BI (posterior probability–below). The BI trees were not represented. The scale bar represents the average nucleotide substitutions per site. Brown arrow shows the Capitella capitata from type-locality (west Greenland). One asterisk (*) indicates that only COI sequence was used and two asterisks (**) indicate that only 16S sequence was used. A tilde (~) indicates that the support value was lower than 70 (ML) or 0.7 (BI), and a dash (-) indicates that the branch was not recovered. (TIF) [file pone.0177760.s002.tif]
