# Supplementary material for: Demystifying the Capitella capitata complex (Annelida, Capitellidae) diversity by morphological and molecular data along the Brazilian coast
Source: PLoS One. 2017 May 31;12(5):e0177760. doi: 10.1371/journal.pone.0177760 (PMC5451021; doi:10.1371/journal.pone.0177760)
Supplement: S1 Table — Intraspecific (in bold) and interspecific mean pairwise genetic distances based on p-distance for 16S (on top) and COI (below). All values are in percentage. The number in parenthesis represents the standard error. (DOCX) [file pone.0177760.s003.docx]

**S1 Table.** **Intraspecific (in bold) and interspecific mean pairwise genetic distances based on *p*-distance for 16S (on top) and COI (below).** All values are in percentage. The number in parenthesis represents the standard error.

|  | 1 | 2 | 3 | 4 | 5 |
| --- | --- | --- | --- | --- | --- |
| 1 - *C*. *nonatoi* sp. n. | **0.4 (0.2)** |  |  |  |  |
|  | **1.5 (0.5)** |  |  |  |  |
| 2 - *C*. *neoaciculata* sp. n. | 21.3 (2.3) | **0.5 (0.2)** |  |  |  |
|  | 18.4 (2.3) | **1.6 (0.5)** |  |  |  |
| 3 - *C*. *biota* sp. n. | 25.7 (2.4) | 28.3 (2.5) | **0.4 (0.2)** |  |  |
|  | 15.7 (2.2) | 17.8 (2.2) | **2.2 (0.6)** |  |  |
| 4 - *C*. *aracaensis* sp. n. | 26.8 (2.4) | 34.2 (2.7) | 26.4 (2.4) | **0.0 (0.0)** |  |
|  | 17.8 (2.4) | 14.1 (2.2) | 17.7 (2.4) | **0.0 (0.0)** |  |
| 5 - *C*. *capitata* | 21.5 (2.3) | 23.8 (2.3) | 25.6 (2.5) | 28.0 (2.5) | **nd** |
|  | 21.8 (2.6) | 16.7 (2.2) | 19.7 (2.4) | 18.4 (2.4) | **1.7 (0.6)** |
